# Gender inequity and age-appropriate immunization coverage in India from 1992 to 2006

**DOI:** 10.1186/1472-698X-9-S1-S3

**Published:** 2009-10-14

**Authors:** Daniel J Corsi, Diego G Bassani, Rajesh Kumar, Shally Awasthi, Raju Jotkar, Navkiran Kaur, Prabhat Jha

**Affiliations:** 1 Centre for Global Health Research, St. Michael's Hospital, University of Toronto, Toronto, ON, M5C 1N8, Canada,; 2 Population Health Research Institute, McMaster University, Hamilton, ON, L8L 2X2, Canada,; 3 School of Public Health, Postgraduate Institute of Medical Education and Research, Chandigarh, India,; 4 Department of Pediatrics, King Georges Medical University, Lucknow, Uttar Pradesh, India

## Abstract

**Background:**

A variety of studies have considered the affects of India's son preference on gender differences in child mortality, sex ratio at birth, and access to health services. Less research has focused on the affects of son preference on gender inequities in immunization coverage and how this may have varied with time, and across regions and with sibling compositions. We present a systematic examination of trends in immunization coverage in India, with a focus on inequities in coverage by gender, birth order, year of birth, and state.

**Methods:**

We analyzed data from three consecutive rounds of the Indian National Family Health Survey undertaken between 1992 and 2006. All children below five years of age with complete immunization histories were included in the analysis. Age-appropriate immunization coverage was determined for the following antigens: bacille Calmette-Guérin (BCG), oral polio (OPV), diphtheria, pertussis (whooping cough) and tetanus (DPT), and measles.

**Results:**

Immunization coverage in India has increased since the early 1990s, but complete, age-appropriate coverage is still under 50% nationally. Girls were found to have significantly lower immunization coverage (p<0.001) than boys for BCG, DPT, and measles across all three surveys. By contrast, improved coverage of OPV suggests a narrowing of the gender differences in recent years. Girls with a surviving older sister were less likely to be immunized compared to boys, and a large proportion of all children were found to be immunized considerably later than recommended.

**Conclusions:**

Gender inequities in immunization coverage are prevalent in India. The low immunization coverage, the late immunization trends and the gender differences in coverage identified in our study suggest that risks of child mortality, especially for girls at higher birth orders, need to be addressed both socially and programmatically.

**Abstract in Hindi:**

See the full article online for a translation of this abstract in Hindi.

## Abstract in Hindi

See Additional file 1 for a translation of the abstract to this article in Hindi.

## Background

In India there is a well-documented history of son preference [1-4]. A growing body of literature has examined the impacts of India's son preference on child survival, juvenile sex ratio, and the numbers of "missing" women [5-10]. There is evidence that the son preference in India and other South Asian countries contributes to disadvantage in women's health throughout the life course [11]. Disadvantage for girls in India begins with a reduced chance of being born at all, and those who are born face lower access to preventive care and treatment of disease compared to boys [6,11,12].

Girls born in India have a 40% greater risk of ill health compared to boys and are less likely to access health care, in particular immunization [11,13,14]. Boys, however, are more likely than girls to die in the first month of life from perinatal conditions, such as birth asphyxia and birth trauma. Only two other conditions (unintentional injuries and congenital anomalies) are more common among boys than girls. Beyond these causes, and contrary to the trends observed in most of the world, in India more girls than boys die of acute respiratory diseases, infectious and parasitic diseases, and viral infections [15,16].

Many of the deaths among India's children are avoidable, including those from the childhood cluster of vaccine preventable diseases (especially measles and tetanus), malaria, diarrhoea caused by organisms such as rotavirus, and acute respiratory infections caused by *Streptococcus pneumonia* and *HÃ¦mophilus influenza* type b (HiB). Recent data shows that immunization - long established worldwide as a highly cost-effective lifesaver - still reaches only a minority of India's children, even after the substantial improvements in vaccination coverage against measles and polio. To make matters worse, girls are especially vulnerable due to inequities in access to immunization coverage [17].

Preference for sons in India has been noted to vary across regions, levels of fertility, and order of birth [1,18-20]. A wide variety of studies have examined the affects of India's son preference on child mortality and India's sex ratio in light of changing fertility patterns and concern for "missing" women. Less research has focused on the influence of India's son preference on gender inequities in access to health care, specifically immunization, and how this may have varied with time and across regions. In this article we investigate the presence of gender inequities in terms of access to timely immunization coverage. We will focus on trends in gender inequities at the national level, by birth order, and by state of residence using data collected from 1992 to 2006.

### Previous research

Inequities in immunization coverage by gender have been shown to exist throughout India [12]. Of the 17 major states, 10 have demonstrated inequity in full immunization coverage against girls. Even states that perform well in immunization coverage struggle with considerably different immunization rates between boys and girls [20]. A search of available literature yielded several studies reporting lower immunization coverage among girls as compared to boys. A study of more than 4000 rural Indian children in 1993-1994 indicated that fewer than 55% of children were fully vaccinated and that girls had a 5% lower coverage compared to boys [17]. In 1992, Bonu et al evaluated vaccination coverage among children aged 12-35 months before and after a three-year government vaccination-awareness program in rural areas of four north Indian states. Prior to the program, girls were found to be at a disadvantage compared to boys and the differences in coverage by gender persisted following the program's completion [21]. Other studies reviewed indicated lower immunization coverage for girls compared to boys, although differences were non-significant [22,23].

**Figure 1:**
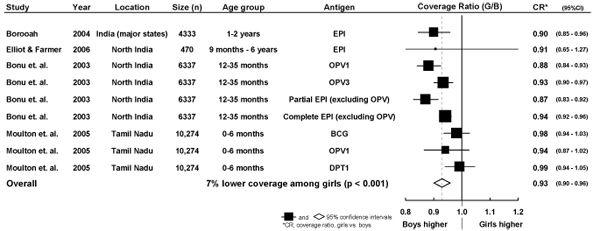
**Girl-to-boy ratios of immunization coverage and combined estimates derived from previously published studies.** The girl-to-boy immunization coverage ratios are based on the results of studies where data was available for calculation of pooled estimates. A CR of less than 1.0 indicates higher coverage in favour of boys.

We compiled data from these four studies comprising nine sub-samples (based on a combination of different age groups and antigens) to obtain an overall ratio of coverage (girls vs. boys). We observed an overall coverage ratio estimate of 0.93 (95% CI: 0.90, 0.9, Figure 1) - indicating that among these studies girls were 7% less likely to be immunized when compared to boys (p<0.001).

## Methods

### Data sources

This study uses data from three consecutive rounds of the Indian National Family Health Survey (NFHS) [24-26]. The International Institute for Population Sciences coordinated each round of the survey with support from several international organizations. The three cross-sectional surveys were conducted during 1992-93, 1998-99 and 2005-06. A summary of the coverage and target population for each round is presented in Table 1. The sampling, questionnaire structure, and content of the NFHS surveys follow what has been adopted by the Demographic Health Surveys (DHS) in other developing countries. The NFHS uses nationally representative area-based sampling frames in each survey [27]. The NFHS produced high response rates in all states. Details of the survey methodology and response rates have been published for each round of the survey [24-26].

**Table 1:** Overview of India's National Family Health Survey (NFHS).

	Survey Phase		
	
	1992-1993	1998-1999	2005-2006
	(NFHS-1)	(NFHS-2)	(NFHS-3)
Sample size (women)	89,777	91,000	230,000
Age group (women)	13-49	15-49	15-49
Data collection period			
Start	April 1992	Nov 1998	Dec 2005
Finish	Sept 1993	Dec 1999	Aug 2006
Number of states	25	26	29
Living cohorts	1988-1993	1996-1999	2001-2006
Total sample (living	45,275	30,821	48,468
children under 5)			
Sample included in analysis	43,732	29,669	47,709
Reference period for			
immunization coverage			
Start	Jan 1988	Jan 1996	Jan 2001
Finish	Aug 1993	Dec 1999	Aug 2006
			

### Sample for analysis

Our sample for analysis includes all children below five years of age with complete immunization histories (*N* = 121,100). The 1998-1999 survey only included children up to 35 months of age at the time of the survey. About 3% of children were excluded due to missing data on immunization coverage. Total sample sizes of children under five along with analysis samples for each round of the NFHS are detailed in Table 1.

### Indicators and measures

We defined immunization coverage as up to date, age-appropriate immunization coverage. Standard indicators of immunization coverage are based on the percentage of children who have accumulated the required number of vaccines by a certain age, regardless of timeliness. Age-appropriate vaccination has been shown to be an important component of infection control [28,29] by reducing transmissibility in susceptible populations [30,31] and by increasing the probability of survival [32]. We determined age-appropriate immunization coverage for each antigen using a combination of data from the child's immunization card and maternal recall when cards were unavailable. Previous studies have demonstrated that maternal recall can be a robust estimation of immunization coverage in settings where complete records are not available [33].

**Figure 2:**
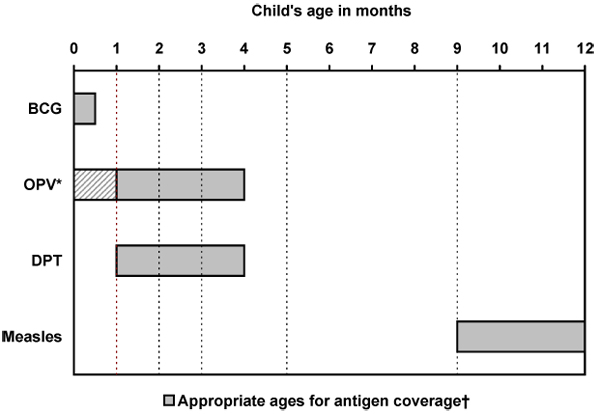
**Recommended expanded program of immunization (EPI) schedule for India.** *OPV-0 is an additional dose of polio given at birth, but is not part of India's national immunization program. ✝Dashed lines indicate ages (1,2,3,5, and 9 months) used in determining appropriate immunization coverage within the child's first year. Children were considered to have age-appropriate EPI coverage if they had received all antigens recommended for their age.

Immunization information was available for the following antigens: bacille Calmette-Guérin (BCG), oral polio vaccine (OPV), diphtheria, pertussis (whooping cough) and tetanus (DPT) vaccine, and measles vaccine. We considered children age-appropriately immunized if they had received all immunizations for their age according to the WHO's Expanded Program on Immunization (EPI) immunization schedule. Modelled on the WHO guidelines, the government of India's Universal Immunization Program (UIP) was introduced in 1985 and includes one dose of BCG (at birth), three doses of OPV and DPT (at 6, 10, and 14 weeks), and one dose of measles (at nine months) [34]. India's EPI/UIP schedule used for our age-appropriate classification is detailed in Figure 2.

Using the child's age in months and the EPI schedule, a composite binary variable indicating EPI complete (yes or no) was created to represent the overall age-appropriate immunization status of each child as follows:

**0-1 month:** child was considered age-appropriately immunized (EPI=1) if they had received BCG;

**2-3 months:** child was considered age-appropriately immunized (EPI=1) if they had received BCG and two doses of OPV and DPT (one dose of OPV and DPT if aged two months);

**4-8 months:** child was considered age-appropriately immunized (EPI=1) if they had received BCG and three doses of OPV and DPT;

**9 months and older:** child was considered age-appropriately immunized (EPI=1) if they had received BCG, three doses of OPV and DPT, and one dose of measles.

To study the influence of birth order and gender of the older siblings, we calculated the birth order and gender of each child in relation to the birth order and gender of their siblings. In a subset of children for whom the complete date of birth (day, month, and year) was known and complete date of immunization was recorded on the immunization card, we calculated age of vaccine receipt for all EPI antigens.

### Data analysis

The analyses in this paper are primarily descriptive and present gender differences in immunization coverage by antigen, birth order, year of birth, and state across the three rounds of the NFHS survey. Using the composite EPI age-appropriate variable, we examined gender differences in coverage by birth order and sibling composition. We also examined the change in age-appropriate coverage by birth cohort (based on year of birth) for each EPI antigen. Births that occurred near to the time of the survey are excluded from the cohort analyses in order to prevent underestimates of coverage due to reduced opportunity to receive complete immunizations among these children. We instead report proportion of children not immunized for these birth cohorts. Gender differences in immunization coverage are presented at the state and national level. Sampling weights were used for all analyses. We tested differences between proportions using *t* statistics. Data were managed and analyzed using Stata (version 10) statistical software [35].

## Results

### Gender inequities in age-appropriate immunization coverage

**Figure 3:**
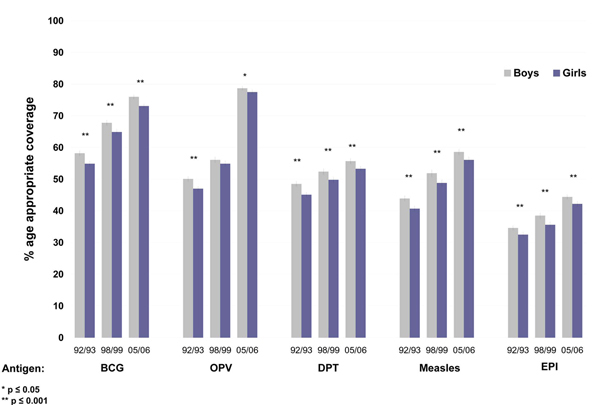
**Percent of children with age-appropriate coverage, by gender, antigen, and date of survey.** Period of NFHS Surveys: 92-93 (NFHS 1), 98-99 (NFHS 2), and 05-06 (NFHS 3). Vertical bars represent 95% confidence intervals (CI).

Immunization coverage has increased in India since 1992-1993 (Figure 3), but age-appropriate EPI coverage remains below 50% nationally for both boys and girls. Coverage of OPV has improved substantially according to the 2005-2006 data, but progress has not been as marked for DPT and Measles.

At the national level, age-appropriate BCG coverage among boys increased from 58.2% (95%CI 57.4; 59.0) in 1992-1993 to 76.0% (95%CI 75.3; 76.7) in 2005-2006. BCG coverage for girls increased from 54.9% (95%CI 54.1; 55.7) in 1992-1993 to 73.1% (95%CI 72.3; 73.8) in 2005-2006. Differences in BCG coverage between girls and boys indicate that girls still have lower access to BCG at the national level (p<0.001, in all three periods).

The percent of boys with age-appropriate OPV coverage increased from 50.1% (95%CI 49.3; 50.8) in 1992-1993 to 78.7% (95%CI 78.1; 79.4) in 2005-2006 and from 47.0% (95%CI 46.2; 47.8) in 1992-1993 to 77.5% (95%CI 76.8; 78.2) in 2005-2006 for girls. The most dramatic increase in age-appropriate OPV coverage has happened between 1998-99 and 2005-2006, but comparison of the coverage for boys and girls at the national level still shows significantly lower OPV coverage (p=0.012) among girls.

Coverage of DPT has shown less improvement. According to the 1992-1993 data, age-appropriate DPT coverage among boys was 48.5% (95%CI 47.7; 49.3), reaching 52.4% (95%CI 51.4; 53.3) in 1998-1999 and climbing only a few percent points in 2005-2006 to 55.7% (95%CI 54.9; 56.5). Coverage for girls has increased from 45.1% (95%CI 44.3; 46.0) in 1992-1993 to 49.8% (95%CI 48.8; 50.7) and to 53.3% (95%CI 52.5; 54.2) in 1998-1999 and 2005-2006, respectively. Girls, however, are experiencing significantly lower coverage rates compared to boys (p<0.001, in all three periods).

Measles, an antigen that requires over 95% coverage to stop transmission in a population, still has very low coverage rates in India. For boys, there has been an increase in age-appropriate coverage from 43.9% (95%CI 43.0; 44.8) in 1992-1993 to 58.6% (95%CI 57.8; 59.5) in 2005-2006. Age-appropriate coverage among girls increased from 40.7% (95%CI 39.9; 41.6) in 1992-1993 to 56.1% (95%CI 55.2; 57.0) in 2005-2006. Differences in coverage by gender are still significant (p<0.05) with girls experiencing lower coverage than boys. Large increases can still be made in the overall levels of coverage of measles vaccination.

As can be noted from the gender specific coverage rates reported above, gender differences in vaccination coverage are consistent and significant, but do not appear to have increased over time. Girls' coverage for every antigen lags behind boys' coverage in all years, but neither gender displays acceptable age-appropriate coverage levels for any antigen (Figure 3).

### Inequities in age-appropriate immunization coverage due to birth order

**Figure 4:**
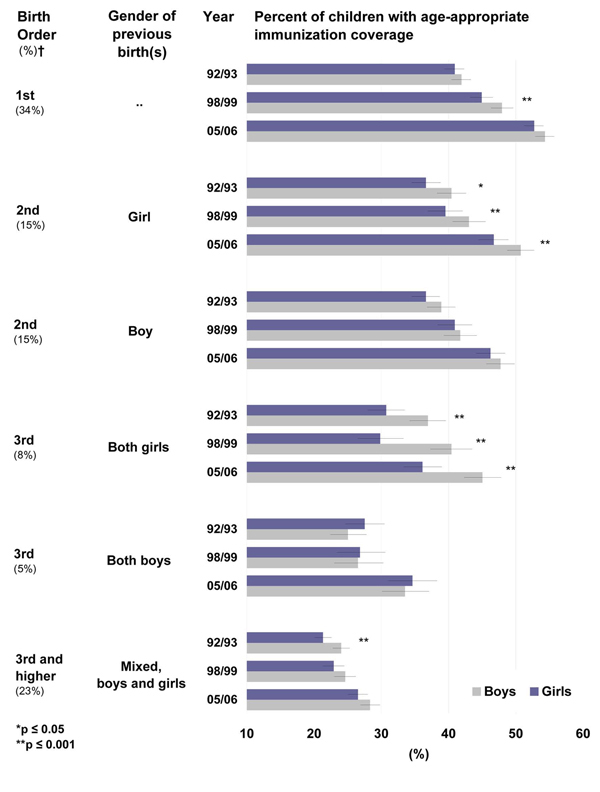
**Percent of children with age-appropriate coverage of EPI vaccines, by birth order, gender of previous birth(s), and survey period.** Overall (EPI) age-appropriate immunization coverage for girls and boys at each birth order (column 1) and sibling gender composition of previous birth(s) (column 2) is represented by the bar chart for each year of the survey period (column 3). ✝Percentages in brackets (column 1) are the proportion of all births represented by each category of birth order and sibling gender in the 2005-6 survey. Horizontal bars represent 95% CI.

Birth order and family composition is an important predictor of vaccination coverage. Higher birth order is associated with a lower probability of being age-appropriately immunized. Despite the increase in rates of age-appropriate immunization coverage over time, the gender gap has not been reduced. Girls are much less likely to be up to date with their immunization at any given age. Furthermore, the gap increases with birth order unless a girl has an older brother. Girls who are born third to a family with two other girls are in the extreme of immunization disadvantage, when compared to boys who are born third to families with two older girls. Only 36.1 % (95%CI 33.3; 39.0) of third born girls with two older sisters are appropriately immunized for their age compared to 45.0% (95%CI 42.3; 47.8, Figure 4) of the third order boys with two older sisters. Higher birth order children (third or higher) with mixed gender sibling (i.e. brothers and sisters) composition have very low age-appropriate immunization coverage, not reaching 30%. Still, girls are experiencing lower coverage rates compared to boys.

This trend is inverted for third order births following two older boys (Figure 4). In this situation, the gender gap is not as pronounced. Like all children at higher birth orders, these children are vulnerable to lower age-appropriate immunization coverage. Indeed, the coverage rates for these children are lower in 2005-2006 than rates observed for first order children born more than 10 years before (i.e. in 1992-1993, Figure 4).

### Trends in gender inequities over time

**Figure 5:**
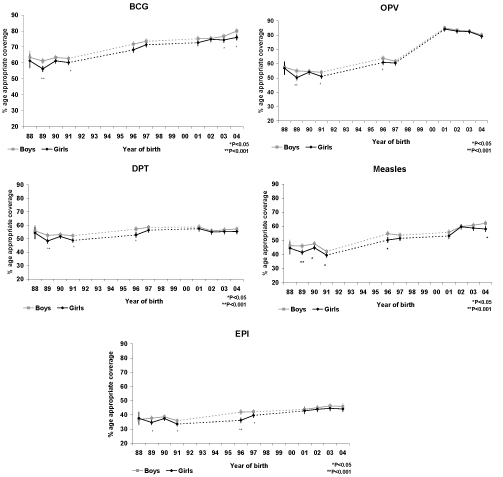
**Trends in age-appropriate immunization coverage of EPI antigens in India by antigen, gender, and year of birth.** Years of birth covered by NFHS surveys are indicated by solid points and lines. Dashed lines indicate periods where data is not available. Vertical bars represent 95% CI.

**Figure 6:**
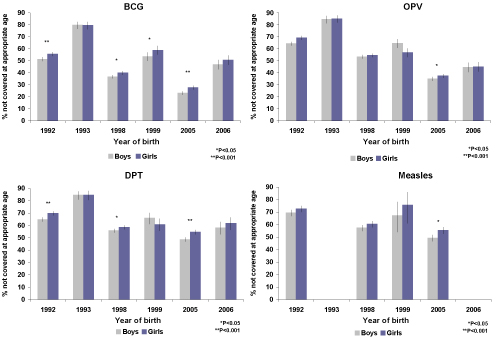
**Percentage of children without age-appropriate immunization coverage in India at the time of each NFHS survey, by antigen and gender.** We calculated the percentage of children born during each survey period who were not appropriately covered for each antigen according to their age. Vertical bars represent 95% CI.

We present trends in age-appropriate immunization coverage by birth cohort and antigen in Figure 5. Age-appropriate immunization by gender is presented for children born during the years between 1988 and 1993, between 1996 and 1999, and between 2001 and 2006 inclusive. For children born during the survey years (1992-93 for NFHS-1, 1997-98 for NFHS-2, and 2005-6 for NFHS-3), we present the proportion of children not age-appropriately immunized in Figure 6.

Age-appropriate coverage of BCG increased over the cohorts analysed for both boys and girls. In the majority of cohorts, girls received lower age-appropriate BCG coverage than boys. This difference remained significant for children born in 2003 and 2004 (p<0.05), Figure 5. For children where the date of immunization was recorded, the median age of receiving BCG decreased from 11.3 weeks in 1990 to 7.0 weeks in 1996 and to 4.7 weeks in 2003. For boys the decrease has been from an age of 11.6 weeks in 1990 to 6.7 weeks in 1996 and to 4.6 weeks in 2003. The WHO/EPI recommendation is that BCG be administered at birth or first contact with health services. Our results indicate a considerable delay in BCG coverage for both boys and girls (see web appendix in Additional file 2). The proportion of boys and girls born during the survey years who were not vaccinated are presented in Figure 6. These estimates are cross-sectional and should be interpreted taking into account future opportunity for immunization.

Age-appropriate coverage of OPV has improved from 1988 to 2004 and similar coverage for both boys and girls has been achieved in recent birth cohorts. Where information is available, it is noted that the age of receiving OPV-1 has been reduced from a median of approximately 13 weeks in 1990 (boys and girls) to less than nine weeks in 2003, near to the recommended age of six weeks. The median time to the second and third polio dose remains above the WHO recommendation of 10 and 14 weeks, respectively (Additional file 2). There has been a reduction in median age for receiving OPV-2 from 18 weeks (for all children) in 1990, to 15 weeks in 2003. The median age to receive OPV-3 was 24 weeks for boys and 33 weeks for girls in 1990. This has been reduced to 21 weeks for boys and 27 weeks for girls in 2003. Girls born in 2003 received OPV-3 more than 13 weeks later than recommended.

Important gender inequities in DPT coverage were noted for children born in 1989, 1991, and 1996. These inequities have not persisted for children born in 2001-4 (Figure 5). Overall levels of DPT coverage have, however, remained below 60% for boys and girls. The median age for receiving the first DPT dose was seven weeks later than recommended for girls and boys in 1990. This has reduced to three weeks later than recommended for children born in 2003 (Additional file 2). Boys and girls born in recent years are also receiving the second and third DPT doses earlier (Additional file 2).

There has been an increase in age-appropriate measles immunization coverage for boys and girls born in 2004 compared with children born in 1988. A difference in coverage between girls and boys is still apparent, even for children born in recent years. Girls born in 2004 had significantly (p<0.05) lower coverage than boys born in the same year. Both girls and boys continue to receive the measles vaccine about six weeks later than recommended (Additional file 2).

### Age at immunization

Across all antigens, the distributions of age at immunization demonstrate significant positive skew (Additional file 2). The children at upper tails of these distributions are being immunized extremely late. This indicates that a sizable amount of children are behind the recommended immunization schedule for all of the evaluated antigens. The median age at immunization appears to be decreasing, suggesting an increase of children being vaccinated on time for those born in recent years. Considerable variability exists in the sample sizes available in each birth cohort due to incomplete data on child's date of birth and/or dates of immunization. There does not appear to be large gender inequities in terms of age at immunization, but a sizeable number of children are behind the recommended immunization schedule for all the evaluated antigens (Additional file 2).

### Gender inequities in immunization coverage by state and region in India

**Figure 7:**
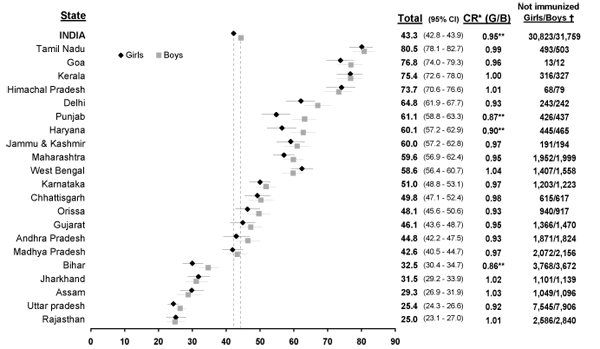
**Percentage of children with age-appropriate EPI coverage, girl-to-boy coverage ratio, and number of children without age-appropriate immunization coverage in India (2005-2006), by state and gender.** NFHS 3 data and state-level sampling weights were used for this figure. ✝Absolute number of children without age-appropriate EPI coverage, estimated from the 2001 Indian Census [34]. *CR, coverage ratio, girls vs. boys **p ≦0.001.

Substantial variation in levels of EPI coverage for boys and girls exists between states in India (Figure 7). Immunization coverage was not strongly correlated with gender inequities in coverage (Pearson's r = 0.06). Six out of the fifteen states that perform above the national average in immunization coverage are also among the eight states with the lowest ratios of girl-to-boy immunization coverage (Figure 7). Nationally, the girl-to-boy ratio of immunization coverage is 0.95 (p<0.001), demonstrating that 5% fewer girls than boys are fully immunized.

The three states with the lowest girl-to-boy ratios of immunization coverage are the northern states of Punjab, Haryana, and Bihar. In these states, between 10 and 14% fewer girls than boys are fully immunized. Other northern states, including Uttar Pradesh and Delhi, demonstrate low girl-to-boy coverage ratios (0.92, and 0.93, respectively) although these differences in coverage are not statistically significant. The southern Indian states of Tamiml Nadu and Keral demonstrate high levels of immunization coverage (80.5% and 75.4%, respectively) and near equity in coverage by gender. Gender inequities in immunization coverage are not clearly determined by a north south divide. Two northern states, Himachal Pradesh and Rajasthan, demonstrate an immunization coverage ratio that favours girls (Figure 7). Additionally, the southern state of Andhra Pradesh demonstrates a large difference in coverage favouring boys (0.93, p=n.s.). The northeastern states of Jharkhand and Assam demonstrate the largest coverage ratios in favour of girls, but are among the states with the lowest coverage rates.

Using proportions of children not appropriately immunized for their age in 2005-6 and population data from the 2001 Census [36], we estimate that, nationally, up to 31M girls and 32M boys aged 0 to four are lacking age-appropriate immunization coverage in India. We present estimated numbers of children lacking age-appropriate coverage for boys and girls aged 0 to four in 21 states in Figure 7. Nationally, and for many states, the absolute number of boys exceeds the number of girls lacking complete age-appropriate immunization coverage. This is due to gender imbalances in India's population structure, leading to fewer girls than boys at these ages [6,36].

## Discussion

Using three nationally representative surveys from India, our findings indicate that, at the national level, girls have lower immunization coverage than boys. We noted that girls at higher birth orders and with older sisters are at greater risk of missing antigens compared to boys of the same birth order and sibling gender composition. Analyses of children by birth year revealed consistently lower coverage of BCG and measles for girls compared to boys, while also suggesting that gender inequities in OPV and DPT coverage may have narrowed for children born in recent years. Gender inequity in immunization coverage was found to be greatest in three northern Indian states, consistent with previous findings of strong son preference in these areas [1,7,19]. The gender inequities in access to preventive care in India noted here are likely to reflect, at least in part, the societal preference for sons in India [11,20].

Our estimates are lower than those published in the NFHS reports as these reports do not account for the timeliness of vaccine delivery [24-26]. Analyses of age at immunization indicated minimal differences between girls and boys for all antigens, suggesting a child's gender may affect the decision to immunize, but not timeliness of coverage.

The use of age-appropriate immunization coverage rates as a health indicator can aid in determining the consequences of not receiving timely vaccination. In our analysis, a substantial number of boys and girls were receiving BCG between one and two years after birth. Studies in the US indicate that the timeliness of immunization coverage can have a considerable impact on child survival [37,38]. In Bangladesh, a study revealed timely BCG immunization could reduce the mortality risk up to 40% for children vaccinated between 60 and 180 days of life and up to 80% for children vaccinated within the first 60 days of life [32]. Similar results have been shown for the effect of age-appropriate DPT immunization and the risk of pertussis [39]. Socioeconomic characteristics associated with delayed immunization have been rarely studied in India [40] and further research is needed in this area.

Our finding that gender inequities in immunization coverage are not correlated with overall immunization coverage is consistent with previous studies [12,20]. Several states with low girl-to-boy coverage ratios (e.g. Punjab, Haryana, and Delhi) are performing above the national average in terms of overall coverage. Data from the DHS in India and other South Asian countries indicate that the gender differences in immunization coverage are observed among all socio-economic levels, with some indication that differences may be larger among the rich than among the poor [12].

Gender inequity in access to health services is believed to be one consequence of larger societal circumstances across South Asia that favour boys and leads to the marginalization of women from a young age [11]. An exploration of socioeconomic and sociocultural determinants of gender inequities in immunization coverage is beyond the scope of this work. Readers are referred to other studies investigating the determinants of gender inequities in sex ratios [6,7], child mortality [5,41,42] fertility patters [1,2], and nutrition [17,20]. Further investigation into the determinants of continuing gender inequities in India's immunization coverage is required.

Along with improved nutrition, immunization is a major tool for saving children's lives, standing out, even in settings of very low income, as a highly cost-effective and efficient intervention [43]. Currently, in India, two-thirds of the children who die of measles and other preventable childhood diseases would have survived if they had had access to immunization. The additional annual per capita cost necessary to reach 90% of Indian children with the six basic vaccines already included in the national immunization program - diphtheria, tetanus, pertussis, TB (BCG), polio, and measles - would be less than three rupees (eight US cents) in the poorest states and even less in wealthier states [44].

Our study shows that current inadequate levels of immunization coverage in India are only part of the problem. Gender inequity in access to health programs is responsible for a considerable number of avoidable deaths. India's states could save even more lives by addressing deep-seated social and cultural issues responsible for gender discrimination at the household level, where girls are seen as a burden and boys as a resource [41]. Campaigns to raise awareness of gender inequities in conjunction with improvement in vaccine delivery strategies with a focus on timeliness of coverage may be a means to that end. Continuing research is needed in order to identify effective social policies of reducing gender inequities in access to immunizations and other health practices across India and other parts of South Asia.

## Conclusions

In India, inequities in girls' access to health services have persisted in recent years. Our research has examined this disparity through a study of gender inequities in child immunization coverage. Young girls are especially vulnerable to these gaps in coverage, but efforts need to be made to increase overall immunization coverage for both girls and boys. Over half of children under five are not fully immunized, and a large proportion of those immunized are being immunized too late. These two factors can lead to increased transmissibility of infections, reducing protective effects of immunization and contributing to avoidable child deaths. In 2005, over 60 million children were not appropriately immunized in India.

## List of abbreviations used

BCG: Bacille Calmette-Guérin; DHS: Demographic Health Surveys; DPT: Diphteria, Pertussis and Tetanus; EPI: Expanded Program on Immunization; Hib: *HÃ¦mophilus influenza* type b; NFHS: Indian National Family Health Survey; OPV: Oral Polio Vaccine; UIP: Universal Immunization Program.

## Competing interests

The authors declare that they have no competing interests.

## Authors' contributions

DC and DB conducted the literature review, data analysis and drafted the manuscript. PJ, RK, SA, RJ, NK, DB, and DC designed and coordinated the study. All authors participated in the interpretation of the results and reviewing the final manuscript.

## Additional material

## Supplementary Material

Additional file 1

Additional file 2 This web appendix presents the distributions (box and whisker plots) of the time to vaccination for each antigen by year of birth. Boys and girls are presented separately. Dashed red lines indicate time of age-appropriate vaccine administration. Red diamonds indicate the mean time to vaccination. The full data table is also presented. Children born in 1994, 1995, 2000 and 2006 (for measles only), are not covered by the NFHS surveys and no data is presented for these years.
