# Supplementary material for: Gender inequity and age-appropriate immunization coverage in India from 1992 to 2006
Source: BMC Int Health Hum Rights. 2009 Oct 14;9(Suppl 1):S3. doi: 10.1186/1472-698X-9-S1-S3 (PMC3226235; doi:10.1186/1472-698X-9-S1-S3)
Supplement: Additional file 1 [file 1472-698X-9-S1-S3-S1.pdf]

भारत में वर्ष 1992 से 2006 तक लैंगिक असमानता व आयु-उपयुक्त टीकाकरण कवरेज।

Daniel J. Corsi, Diego G. Bassani, Rajesh Kumar, Shally Awasthi, Raju Jotkar, Navkiran Kaur, Prabhat Jha

सारांश

पृष्ठभूमी

भारत में पुत्र पसंदता के परिणाम बाल मृत्यु दर, जन्म के समय लिंग अनुपात तथा स्वास्थ्य सेवाओं के उपयोग में पाए जाने वाले लैंगिक अंतरों में देखे गए हैं। विभिन्न प्रकार के अध्ययनों ने इन परिणामों पर विचार किया है । मगर पुत्र पसंदता के कारण टीकाकरण कवरेज में हो रही लैंगिक असमानताओं पर जो प्रभाव पड़ रहे हैं, उन पर बहुत कम अनुसंधान केंद्रित हुए हैं। इस बात पर भी ध्यान नहीं दिया जा रहा कि ये प्रभाव समय, क्षेत्रों के पार तथा सहोदर संघटन के अनुसार बदल गए हो सकते हैं। हम भारत में हो रही टीकाकरण कवरेज में रूझानों के व्यवस्थित परीक्षण को प्रस्तुत करते हैं जिनमें ध्यान का केन्द्र कवरेज में लैंगिक जन्म आदेश व राज्य के अनुसार आई असमानताओं पर होता है।

विधि

हमने वर्ष 1992 से 2006 के बीच में लगातार तीन बार हुए राष्ट्रीय परिवार स्वास्थ्य सर्वेक्षण के डेटा का विश्लेषण किया है। सम्पूर्ण टीकाकरण रिकार्ड सहित पाँच वर्ष से कम आयु के सभी बच्चों को इस विश्लेषण में शामिल किया गया था। आयु-उपयुक्त टीकाकरण कवरेज का निर्धारण निम्नलिखित प्रतिजनों के आधार पर किया गया था :-
बेसाइल कलमेट-गुरीन (बी.सी.जी.), ओरल पोलियो वैक्सीन (ओ.पी.वी.), डिफ्थीरिया, पर्टुसिस (काली खाँसी), टैटनस (डी.पी.टी.) तथा खसरा।

परिणाम

भारत में 1990 के दशक के शुरुआती दौर से टीकाकरण कवरेज में वृद्धि हुई है। लेकिन संपूर्ण, आयु-उपयुक्त कवरेज राष्ट्रीय स्तर पर अभी भी 50 प्रतिशत से कम है। तीनों सर्वेक्षणों के दौरान लड़कों के मुकाबले लड़कियों में बी.सी.जी., डी.पी.टी. तथा खसरे के लिए टीकाकरण कवरेज में उल्लेखनीय कमी पाई गई थी। इसके विपरीत ओ.पी.वी. की कवरेज में बढ़ोत्तरी, हाल ही के वर्षों में लैंगिक असमानताओं के सीमित हो रहे रूप को दर्शाती है। लड़कों के मुकाबले लड़कियों में प्रतिरक्षा करवाने की संभावना बहुत कम पाई गई थी, यदि उनकी बड़ी बहन जीवित थी । यह भी पाया गया कि सभी बच्चों में से बड़ी मात्रा में बच्चों का टीकाकरण सुझाए गए समय के बाद करवाया गया।

निष्कर्ष

टीकाकरण कवरेज में लैंगिक असमानताएँ आज भी भारत में एक समस्या के तौर पर बरकरार हैं। हमारे अध्ययन में टीकाकरण कवरेज में कमियाँ, टीकाकरण रूझानों में देरी तथा कवरेज में लैंगिक असमानताएँ पाई गई हैं। यह कमियाँ खास कर उच्चतर जन्म आदेश की लड़कियों में बाल मृत्युदर के खतरों को दर्शाती हैं, जिन्हें सामाजिक व कार्यक्रम स्तर पर संबोधित करने की ज़रुरत है।
