# Supplementary material for: Gender inequity and age-appropriate immunization coverage in India from 1992 to 2006
Source: BMC Int Health Hum Rights. 2009 Oct 14;9(Suppl 1):S3. doi: 10.1186/1472-698X-9-S1-S3 (PMC3226235; doi:10.1186/1472-698X-9-S1-S3)
Supplement: Additional file 2 — This web appendix presents the distributions (box and whisker plots) of the time to vaccination for each antigen by year of birth. Boys and girls are presented separately. Dashed red lines indicate time of age-appropriate vaccine administration. Red diamonds indicate the mean time to vaccination. The full data table is also presented. Children born in 1994, 1995, 2000 and 2006 (for measles only), are not covered by the NFHS surveys and no data is presented for these years. [file 1472-698X-9-S1-S3-S2.pdf]

Web Appendix:
Distributions of time to immunization, by antigen, gender and year of birth.

Supplement to: Corsi DJ, Bassani DG, Kumar R, et al. Gender inequity and age-appropriate immunization coverage in India from 1992 to 2006.

This web appendix presents the distributions (box and whisker plots) of the time to vaccination for each antigen by year of birth. Boys and girls are presented separately. Dashed red lines indicate time of age-appropriate vaccine administration. Red diamonds indicate the mean time to vaccination. The full data table is also presented. Children born in 1994, 1995, 2000 and 2006 (for measles only), are not covered by the NFHS surveys and no data are presented for these years.

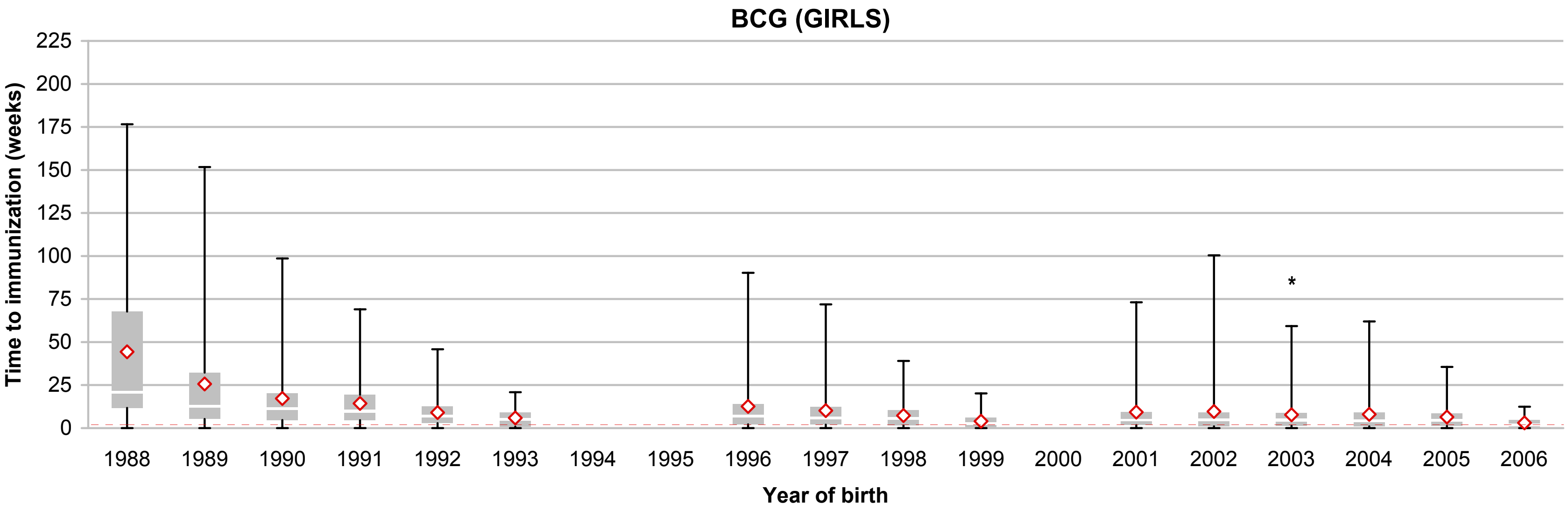

	1988	1989	1990	1991	1992	1993	1994	1995	1996	1997	1998	1999	2000	2001	2002	2003	2004	2005	2006
1st	0.00	0.00	0.00	0.00	0.00	0.00			0.00	0.00	0.00	0.00		0.00	0.00	0.00	0.00	0.00	0.00
25th	11.71	5.29	4.71	4.57	2.86	0.86			2.14	2.00	1.57	0.43		1.71	1.00	1.43	1.00	1.29	0.43
50th	20.86	12.71	11.29	9.86	6.86	5.14			7.00	5.86	5.57	2.57		4.71	4.86	4.71	4.29	4.57	1.86
75th	67.71	32.29	20.29	19.43	12.57	9.14			14.00	12.29	10.29	6.00		9.43	9.00	8.71	9.00	8.57	4.71
99th	176.57	151.71	98.57	69.00	45.86	20.86			90.29	71.86	39.00	20.14		73.14	100.43	59.29	62.00	35.57	12.43
Mean	44.35	25.61	17.26	14.35	9.02	5.88			12.59	10.19	7.35	3.98		9.32	9.66	7.77	8.01	6.46	3.02
SD	47.58	32.36	20.56	14.69	8.83	5.28			18.44	14.21	7.86	4.88		15.00	18.54	`	11.92	7.26	3.14
Minimum	0.00	0.00	0.00	0.00	0.00	0.00			0.00	0.00	0.00	0.00		0.00	0.00	0.00	0.00	0.00	0.00
Maximum	176.57	162.00	131.86	109.86	63.71	20.86			135.00	115.00	83.86	60.43		228.86	166.43	161.43	104.29	67.14	18.71
N	42	435	814	1249	1154	131			546	1081	1341	318		490	844	1008	1349	1780	365

* p <0.05 test for difference in mean time to vaccination between girls and boys

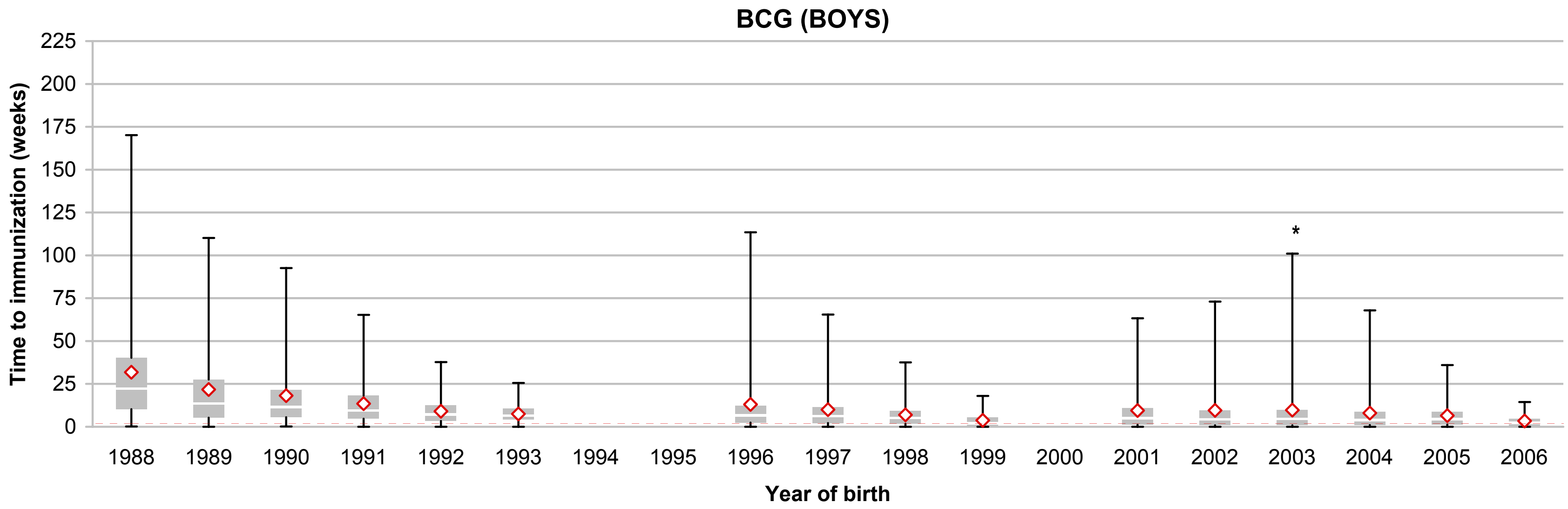

	1988	1989	1990	1991	1992	1993	1994	1995	1996	1997	1998	1999	2000	2001	2002	2003	2004	2005	2006
1st	0.14	0.00	0.14	0.00	0.00	0.00			0.00	0.00	0.00	0.00		0.00	0.00	0.00	0.00	0.00	0.00
25th	10.29	5.29	5.57	4.71	3.43	4.29			2.43	2.14	1.71	0.43		1.14	0.86	0.86	1.00	1.29	0.57
50th	22.29	13.57	11.57	9.43	7.14	6.57			6.71	6.29	5.14	2.00		4.86	4.29	4.57	4.00	4.57	2.29
75th	40.29	27.57	21.57	18.14	12.57	10.71			12.43	11.57	9.43	5.43		10.86	9.57	10.00	8.71	8.71	4.71
99th	170.14	110.14	92.57	65.29	37.71	25.57			113.43	65.43	37.57	18.00		63.29	73.00	101.00	67.86	36.00	14.43
Mean	31.81	21.74	18.20	13.47	9.06	7.50			13.08	9.97	6.92	3.78		9.47	9.53	9.76	7.99	6.53	3.35
SD	36.93	24.24	20.53	13.43	7.88	5.46			20.26	13.14	7.34	5.06		14.29	17.42	17.22	12.83	7.32	3.51
Minimum	0.14	0.00	0.00	0.00	0.00	0.00			0.00	0.00	0.00	0.00		0.00	0.00	0.00	0.00	0.00	0.00
Maximum	170.14	127.71	129.57	108.43	58.00	25.57			140.57	104.00	50.00	64.14		234.14	189.71	153.43	106.00	66.71	22.43
N	51	500	900	1419	1291	150			680	1272	1626	330		511	885	1231	1560	1977	391

* $p < 0.05$ test for difference in mean time to vaccination between girls and boys

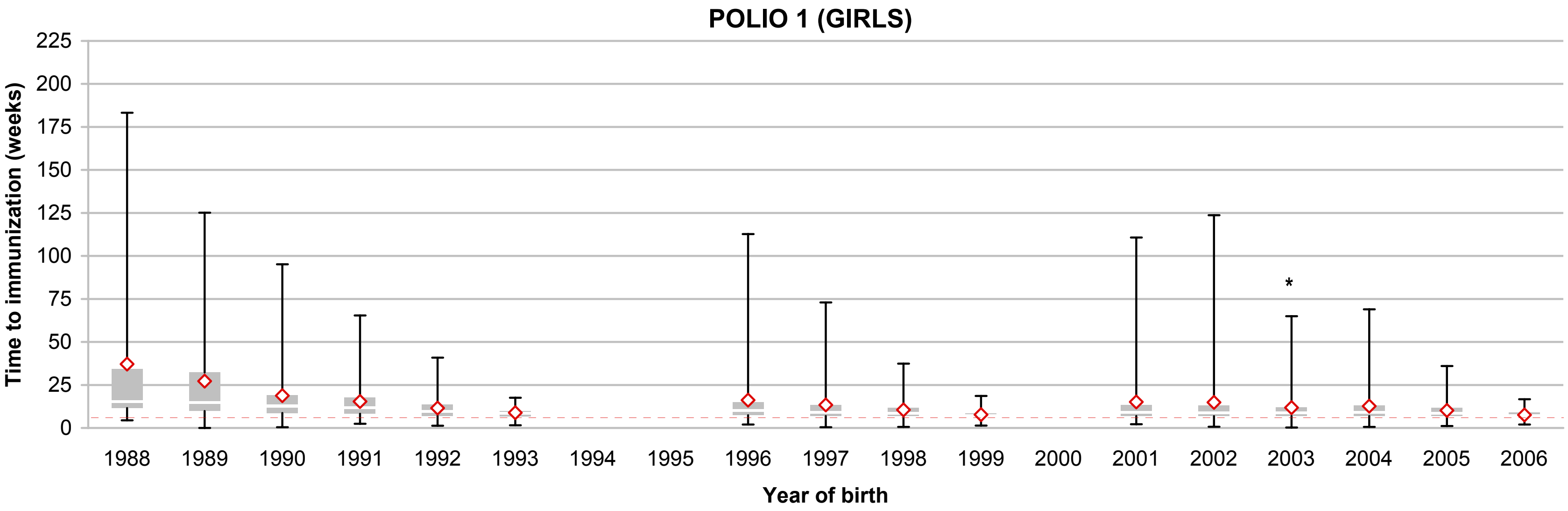

	1988	1989	1990	1991	1992	1993	1994	1995	1996	1997	1998	1999	2000	2001	2002	2003	2004	2005	2006
1st	4.43	0.00	0.43	2.43	1.29	1.57			2.00	0.43	0.57	1.43		2.14	0.71	0.29	0.57	1.14	2.00
25th	11.57	9.86	8.71	8.29	7.14	6.71			7.43	7.00	6.86	6.29		7.00	6.86	7.00	7.00	6.86	6.29
50th	15.29	14.71	12.71	11.71	9.71	8.57			10.14	9.00	8.57	7.14		9.00	8.86	8.86	9.00	8.43	7.00
75th	34.29	32.29	19.00	17.71	13.71	10.00			15.00	13.43	11.86	8.43		13.29	13.14	12.14	13.14	11.71	8.71
99th	183.29	125.14	95.14	65.43	40.86	17.57			112.71	73.00	37.43	18.57		110.71	123.71	65.00	69.00	36.00	16.71
Mean	37.15	27.19	18.75	15.36	11.60	8.90			16.14	13.38	10.49	7.76		15.15	14.84	11.86	12.70	10.18	7.56
SD	45.08	28.75	18.76	11.90	7.22	3.39			18.78	13.86	6.63	3.93		23.44	19.65	12.22	12.17	6.09	2.73
Minimum	4.43	0.00	0.00	0.00	0.14	0.43			0.00	0.00	0.00	0.00		0.14	0.00	0.00	0.00	0.00	0.29
Maximum	183.29	160.29	122.14	95.29	63.71	17.57			126.14	115.00	83.86	60.43		249.00	188.57	147.57	96.86	57.71	19.14
N	49	493	915	1380	1217	114			569	1132	1418	280		510	880	1059	1410	1764	286

* p <0.05 test for difference in mean time to vaccination between girls and boys

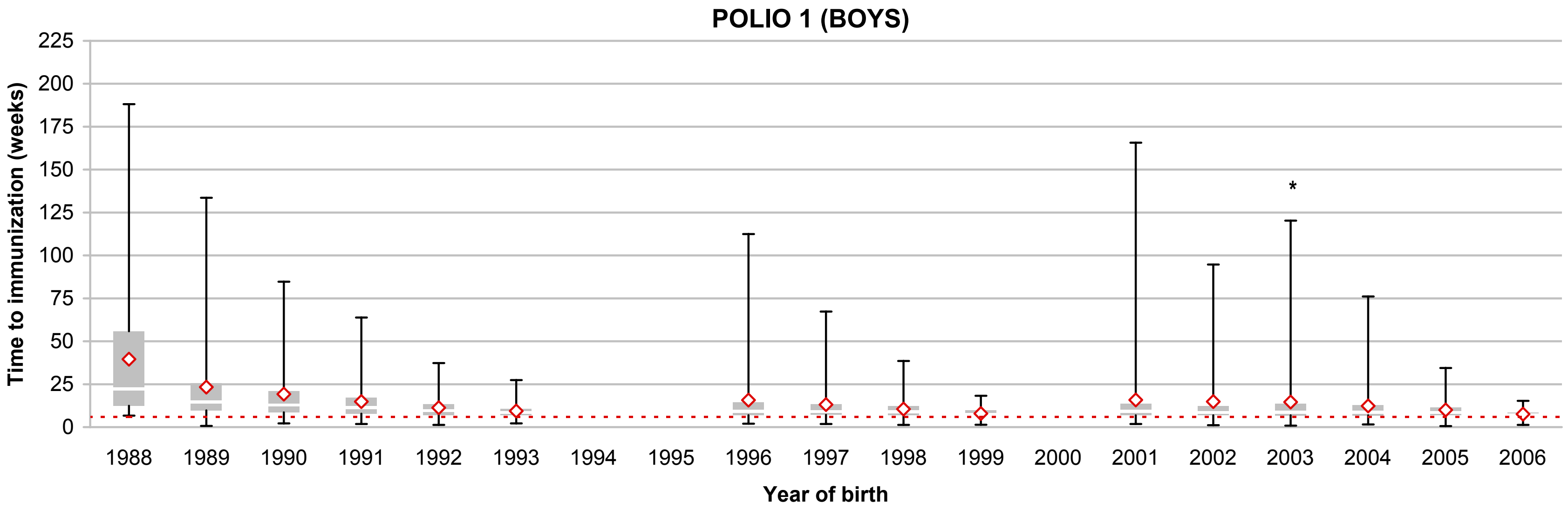

	1988	1989	1990	1991	1992	1993	1994	1995	1996	1997	1998	1999	2000	2001	2002	2003	2004	2005	2006
1st	6.71	0.71	2.14	1.86	1.29	2.14			2.00	1.86	1.29	1.43		1.86	1.14	0.86	1.57	0.57	1.29
25th	12.43	9.71	8.57	7.86	7.14	6.86			7.00	7.14	6.86	6.43		6.86	6.86	6.86	6.86	6.86	6.29
50th	22.29	14.71	12.86	11.29	9.71	8.43			9.14	9.14	8.86	7.29		9.14	8.43	8.71	8.71	8.43	7.14
75th	55.86	25.57	21.00	17.14	13.43	10.57			14.43	13.43	12.29	9.57		13.57	12.29	13.57	12.71	11.29	8.57
99th	188.14	133.57	84.71	63.86	37.29	27.43			112.43	67.29	38.57	18.29		165.71	94.71	120.29	76.14	34.43	15.29
Mean	39.59	23.29	19.22	14.87	11.28	9.37			15.76	13.09	10.54	7.94		15.83	14.87	14.57	12.38	10.00	7.59
SD	44.14	24.67	18.44	11.69	6.52	4.23			19.36	12.41	6.51	3.25		23.74	20.94	18.62	12.32	5.90	2.77
Minimum	4.14	0.43	0.14	0.00	0.14	1.71			0.00	0.00	0.00	0.00		0.57	0.00	0.00	0.00	0.00	0.14
Maximum	188.14	188.43	130.29	98.00	51.29	27.43			141.86	99.14	64.57	38.00		234.29	221.86	153.43	106.00	66.71	18.29
N	67	579	1018	1588	1401	153			704	1325	1677	262		527	928	1280	1615	2013	301

* $p < 0.05$ test for difference in mean time to vaccination between girls and boys

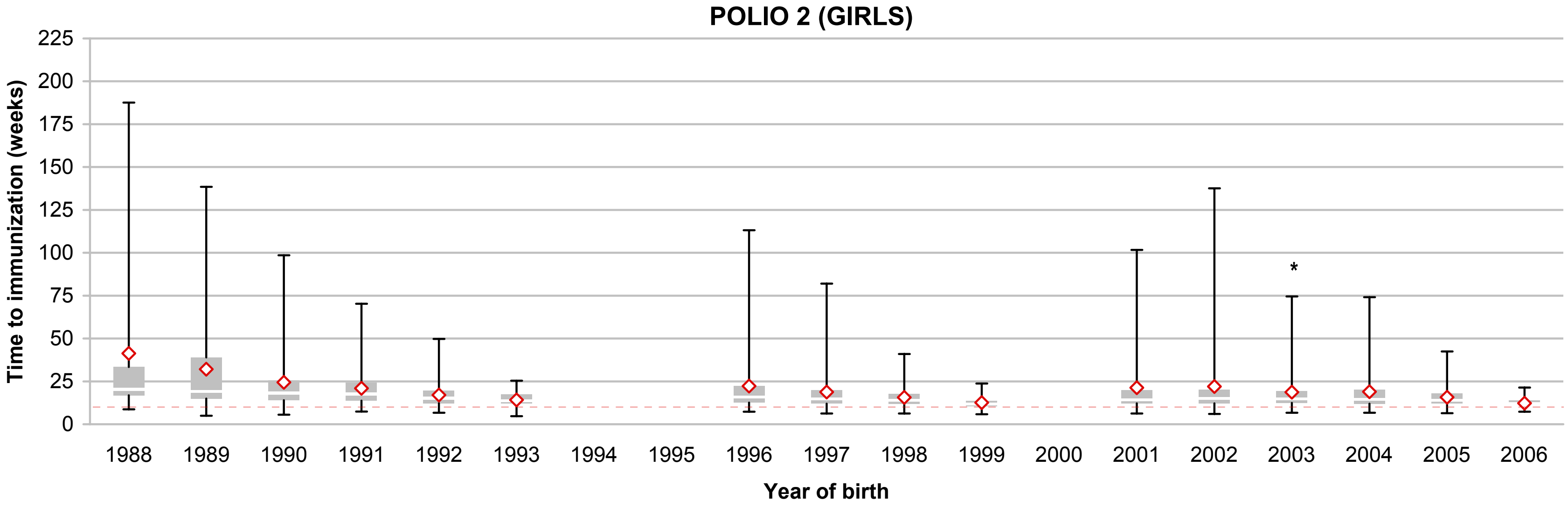

	1988	1989	1990	1991	1992	1993	1994	1995	1996	1997	1998	1999	2000	2001	2002	2003	2004	2005	2006
1st	8.71	5.00	5.57	7.43	6.71	4.71			7.29	6.29	6.29	5.86		6.29	6.00	6.71	6.71	6.43	7.29
25th	16.86	15.00	14.00	13.71	12.29	12.00			12.57	12.00	11.86	10.57		12.14	12.14	12.43	12.00	12.00	11.00
50th	20.43	19.29	18.14	17.57	15.14	13.57			15.86	14.71	14.00	11.86		14.29	15.00	14.86	14.57	14.00	11.43
75th	33.43	39.00	25.57	24.14	19.57	17.29			22.43	19.86	17.71	13.71		20.00	20.14	19.29	20.29	18.00	13.29
99th	187.57	138.43	98.57	70.29	49.71	25.43			113.14	82.00	41.00	23.71		101.71	137.57	74.57	74.14	42.43	21.43
Mean	41.28	32.09	24.40	20.96	17.07	14.19			22.29	18.82	15.65	12.61		21.27	22.00	18.68	18.98	15.75	12.24
SD	44.35	28.96	18.54	12.03	7.64	4.00			19.23	12.94	6.43	5.50		21.29	21.68	13.27	13.00	6.23	2.76
Minimum	8.71	3.71	3.00	2.86	4.14	4.71			4.00	3.14	0.00	5.00		5.29	2.43	4.29	3.86	3.86	6.43
Maximum	187.57	173.57	113.14	99.43	61.71	25.43			134.86	96.71	79.43	69.14		191.00	173.43	152.14	101.71	60.14	24.86
N	49	478	889	1335	1008	65			549	1105	1235	168		498	869	1034	1370	1592	155

* p <0.05 test for difference in mean time to vaccination between girls and boys

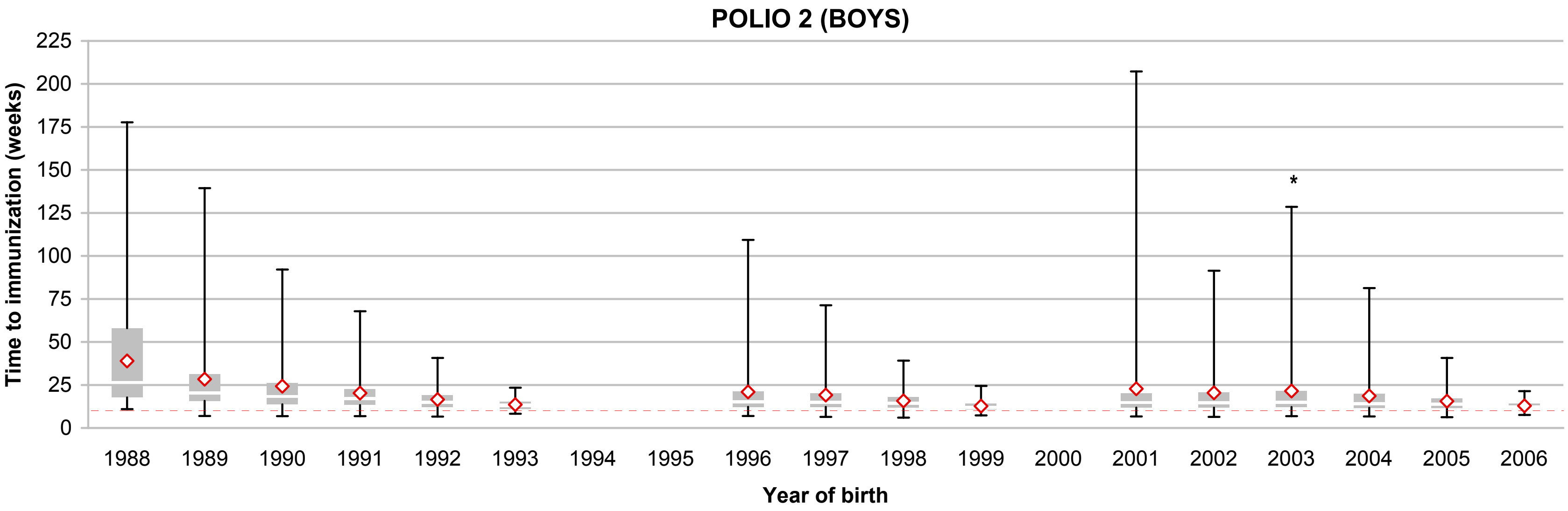

	1988	1989	1990	1991	1992	1993	1994	1995	1996	1997	1998	1999	2000	2001	2002	2003	2004	2005	2006
1st	11.00	7.00	6.86	6.86	6.57	8.29			7.00	6.43	6.00	7.29		6.71	6.43	6.86	6.71	6.29	7.57
25th	17.71	15.71	13.71	13.29	12.14	11.14			12.29	12.29	11.86	10.71		11.86	11.86	12.14	11.71	11.71	11.14
50th	26.29	20.29	18.29	17.00	15.00	13.14			15.14	15.00	14.29	11.86		14.71	14.57	15.00	14.14	13.86	12.14
75th	58.00	31.29	26.14	22.57	19.14	15.14			21.14	20.29	17.86	14.00		20.14	20.57	21.57	19.86	17.29	14.00
99th	177.71	139.43	92.14	67.86	40.71	23.43			109.29	71.29	39.14	24.43		207.29	91.43	128.57	81.29	40.71	21.43
Mean	38.93	28.39	24.24	20.25	16.49	13.53			20.94	19.13	15.78	12.73		22.81	20.47	21.48	18.64	15.60	12.86
SD	32.64	23.63	18.01	11.32	6.58	3.37			18.30	12.65	6.42	4.05		29.93	16.98	20.21	13.16	6.26	2.75
Minimum	11.00	3.43	2.14	1.00	3.71	6.71			4.71	3.29	1.14	6.43		2.43	0.71	1.57	4.43	1.71	6.57
Maximum	177.71	202.71	134.71	109.00	65.00	25.86			146.14	96.29	66.71	48.00		238.29	217.71	158.43	95.86	56.14	26.57
N	62.00	563.00	992.00	1503.00	1140.00	80.00			678.00	1291.00	1474.00	161.00		515.00	917.00	1260.00	1567.00	1836.00	174.00

* $p < 0.05$ test for difference in mean time to vaccination between girls and boys

POLIO 3 (GIRLS)

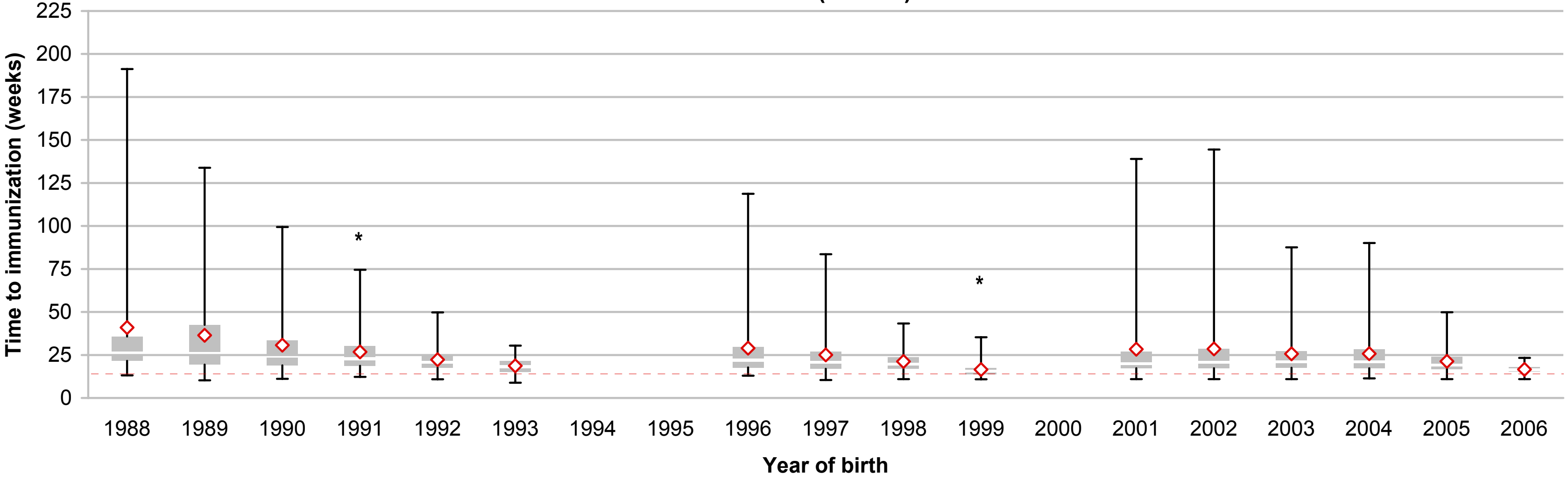

	1988	1989	1990	1991	1992	1993	1994	1995	1996	1997	1998	1999	2000	2001	2002	2003	2004	2005	2006
1st	13.14	10.29	11.14	12.29	10.86	8.86			13.00	10.43	11.00	10.86		11.00	11.00	11.00	11.43	11.00	11.00
25th	21.57	19.43	19.00	18.71	17.57	15.29			17.71	17.00	16.86	13.71		17.29	17.29	17.57	17.29	16.86	15.29
50th	26.14	25.86	24.29	22.86	20.71	18.14			22.00	20.86	19.57	15.71		20.00	20.71	21.00	21.00	19.14	16.29
75th	35.71	42.57	33.43	30.14	24.43	21.43			29.57	27.00	23.71	17.57		27.00	28.57	27.29	28.43	24.00	18.00
99th	191.29	133.86	99.43	74.57	49.71	30.43			118.71	83.57	43.29	35.29		139.00	144.43	87.57	90.14	49.86	23.29
Mean	40.95	36.47	30.74	26.75	22.26	18.71			28.93	25.03	21.20	16.55		28.33	28.54	25.62	25.78	21.34	16.57
SD	37.71	26.97	19.35	13.10	7.36	4.25			19.79	13.59	6.82	6.05		23.40	23.05	14.56	14.48	7.12	2.70
Minimum	13.14	9.43	8.86	6.29	8.29	8.86			9.57	8.43	5.00	10.86		7.14	5.00	8.86	5.57	7.86	10.86
Maximum	191.29	167.57	117.43	105.57	60.57	30.43			127.86	125.86	84.43	73.57		161.14	178.71	142.29	108.14	62.14	25.14
N	45	447	822	1218	759	28			519	1038	1006	80		481	833	977	1313	1327	79

* $p < 0.05$ test for difference in mean time to vaccination between girls and boys

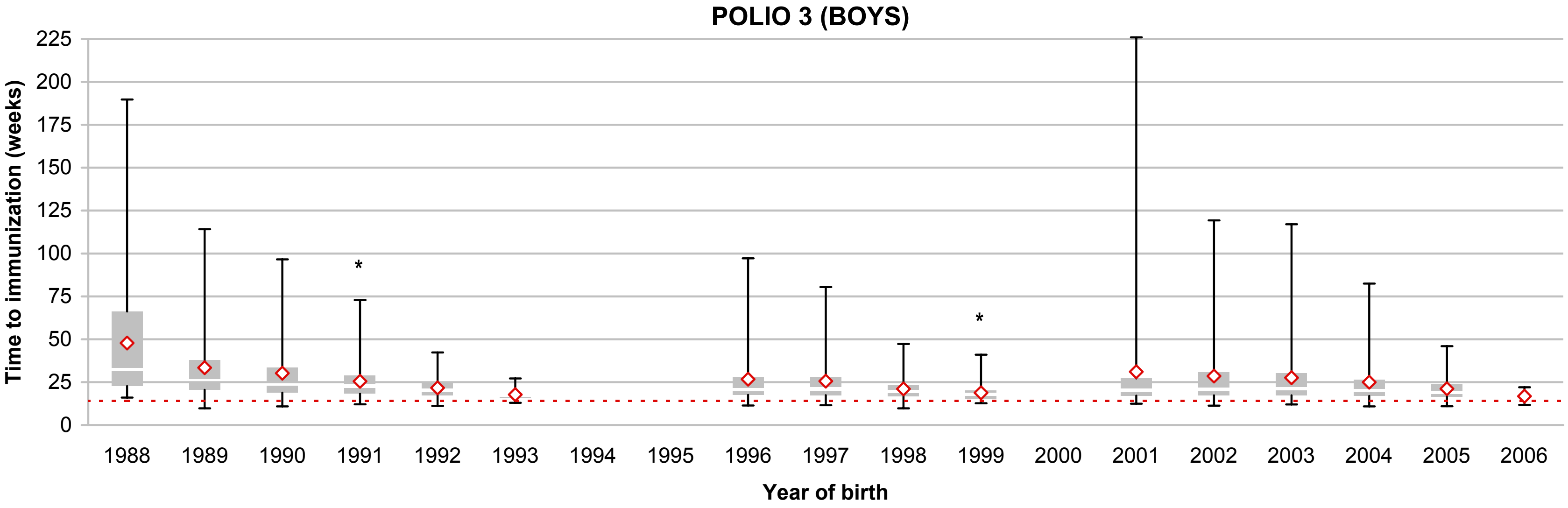

	1988	1989	1990	1991	1992	1993	1994	1995	1996	1997	1998	1999	2000	2001	2002	2003	2004	2005	2006
1st	16.00	9.71	10.86	12.14	11.14	13.00			11.43	11.57	9.71	12.71		12.43	11.29	12.00	10.86	11.00	11.71
25th	22.71	20.57	18.86	18.29	17.43	15.57			17.57	17.43	16.71	15.14		17.14	17.29	17.29	17.00	16.57	15.86
50th	32.29	25.71	24.00	22.57	20.43	17.43			20.57	21.14	19.57	17.86		20.29	20.86	21.29	20.14	19.00	16.86
75th	66.14	37.71	33.57	28.86	24.57	18.43			28.00	27.86	23.43	20.14		27.14	30.86	30.29	26.57	23.86	17.71
99th	189.71	114.14	96.57	72.86	42.29	27.14			97.14	80.43	47.29	41.00		226.00	119.29	117.00	82.43	46.00	22.00
Mean	47.85	33.40	30.18	25.49	21.70	17.73			26.74	25.62	21.09	18.85		31.11	28.55	27.58	25.02	21.20	16.84
SD	35.55	21.88	18.34	11.24	6.42	3.35			17.23	13.88	6.89	6.44		33.99	20.43	18.41	14.10	6.98	1.89
Minimum	16.00	4.29	7.14	0.29	0.71	12.14			10.29	9.29	5.86	12.43		1.29	5.57	1.71	6.86	6.71	11.14
Maximum	189.71	172.71	139.71	101.57	55.29	27.14			137.29	103.00	79.43	67.71		235.14	222.00	157.29	101.14	57.14	25.86
N	61	534	932	1377	873	42			639	1220	1222	84		502	891	1200	1487	1550	80

* p <0.05 test for difference in mean time to vaccination between girls and boys

DPT 1 (GIRLS)

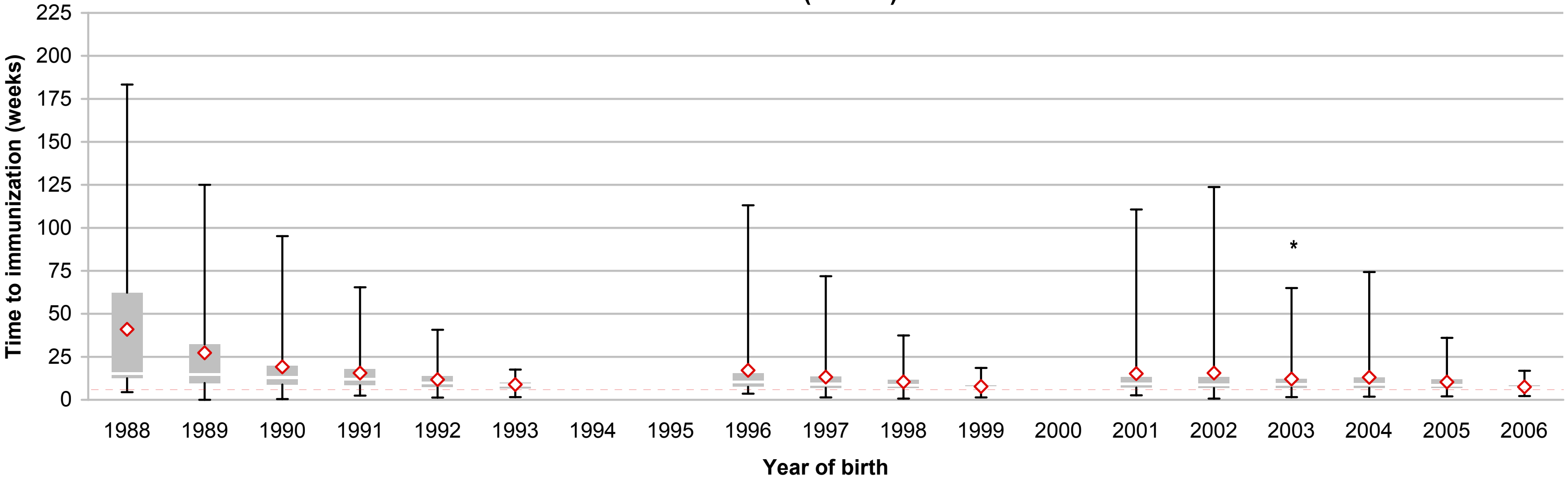

	1988	1989	1990	1991	1992	1993	1994	1995	1996	1997	1998	1999	2000	2001	2002	2003	2004	2005	2006
1st	4.43	0.00	0.43	2.43	1.29	1.57			3.57	1.43	0.71	1.43		2.57	0.71	1.57	1.86	2.00	2.14
25th	12.57	9.71	8.86	8.43	7.29	6.71			7.57	7.00	6.86	6.29		7.14	6.86	7.00	7.00	6.86	6.14
50th	15.29	14.71	13.00	12.00	9.71	8.57			10.29	9.14	8.57	7.14		9.14	8.86	9.00	9.00	8.43	6.86
75th	62.29	32.29	19.86	18.00	13.86	10.00			15.43	13.43	11.71	8.43		13.29	13.29	12.29	13.14	11.86	8.29
99th	183.29	125.00	95.14	65.43	40.71	17.57			113.14	71.86	37.43	18.57		110.71	123.71	65.00	74.29	36.00	16.86
Mean	41.02	27.34	19.15	15.53	11.70	8.89			17.17	13.25	10.47	7.78		15.22	15.56	12.09	13.08	10.32	7.48
SD	48.63	29.59	19.04	11.93	7.20	3.39			19.98	13.42	6.58	3.88		23.33	20.86	12.37	12.84	6.11	2.75
Minimum	4.43	0.00	0.00	0.00	0.14	0.43			0.00	0.00	0.00	0.14		0.71	0.00	0.00	0.00	0.00	0.29
Maximum	183.29	160.29	125.57	95.29	63.71	17.57			126.14	115.00	83.86	60.43		249.00	188.57	138.43	101.14	57.71	18.71
N	50	500	919	1388	1226	115			578	1142	1440	281		513	888	1064	1429	1796	282

* $p < 0.05$ test for difference in mean time to vaccination between girls and boys

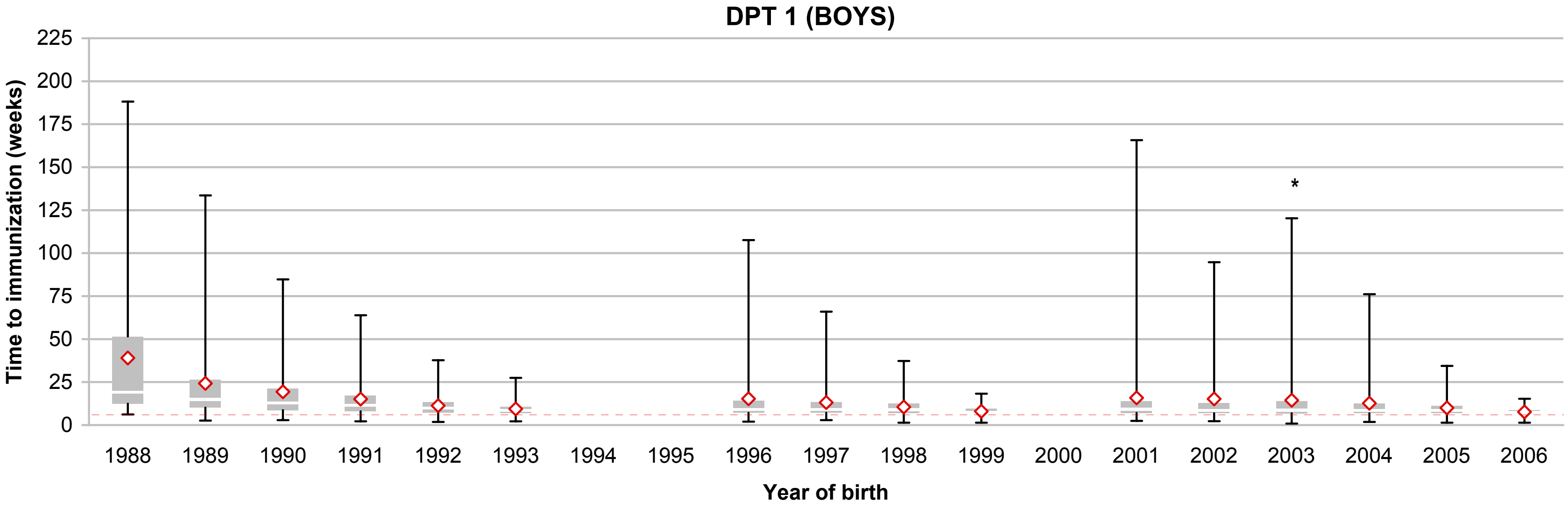

	1988	1989	1990	1991	1992	1993	1994	1995	1996	1997	1998	1999	2000	2001	2002	2003	2004	2005	2006
1st	6.14	2.57	2.86	2.14	1.86	2.14			2.00	2.86	1.43	1.43		2.43	2.29	0.86	1.86	1.43	1.43
25th	12.43	10.14	8.71	8.00	7.14	6.86			7.14	7.14	6.86	6.43		7.00	6.86	6.86	6.86	6.86	6.43
50th	19.14	14.86	13.00	11.57	9.86	8.43			9.14	9.14	8.86	7.29		9.14	8.57	8.71	8.57	8.43	7.14
75th	51.57	26.29	21.29	17.29	13.43	10.57			14.29	13.43	12.43	9.57		13.86	12.86	13.86	12.71	11.14	8.57
99th	188.14	133.57	84.71	63.86	37.71	27.43			107.57	66.00	37.29	18.29		165.71	94.71	120.29	76.14	34.43	15.29
Mean	38.98	24.23	19.39	15.09	11.31	9.36			15.30	13.09	10.51	7.96		15.84	15.24	14.44	12.68	9.99	7.68
SD	44.49	25.65	18.43	11.60	6.53	4.21			18.38	12.10	6.36	3.29		23.47	21.58	18.41	12.75	5.84	2.68
Minimum	4.14	0.71	0.43	0.00	0.14	1.71			0.00	0.00	0.00	0.00		0.57	0.00	0.00	0.00	0.00	0.57
Maximum	188.14	188.43	130.29	98.00	62.00	27.43			141.86	99.14	64.57	38.00		234.29	221.86	153.43	106.00	66.71	18.29
N	67	582	1020	1592	1407	156			706	1338	1700	266		533	943	1292	1632	2040	297

* p <0.05 test for difference in mean time to vaccination between girls and boys

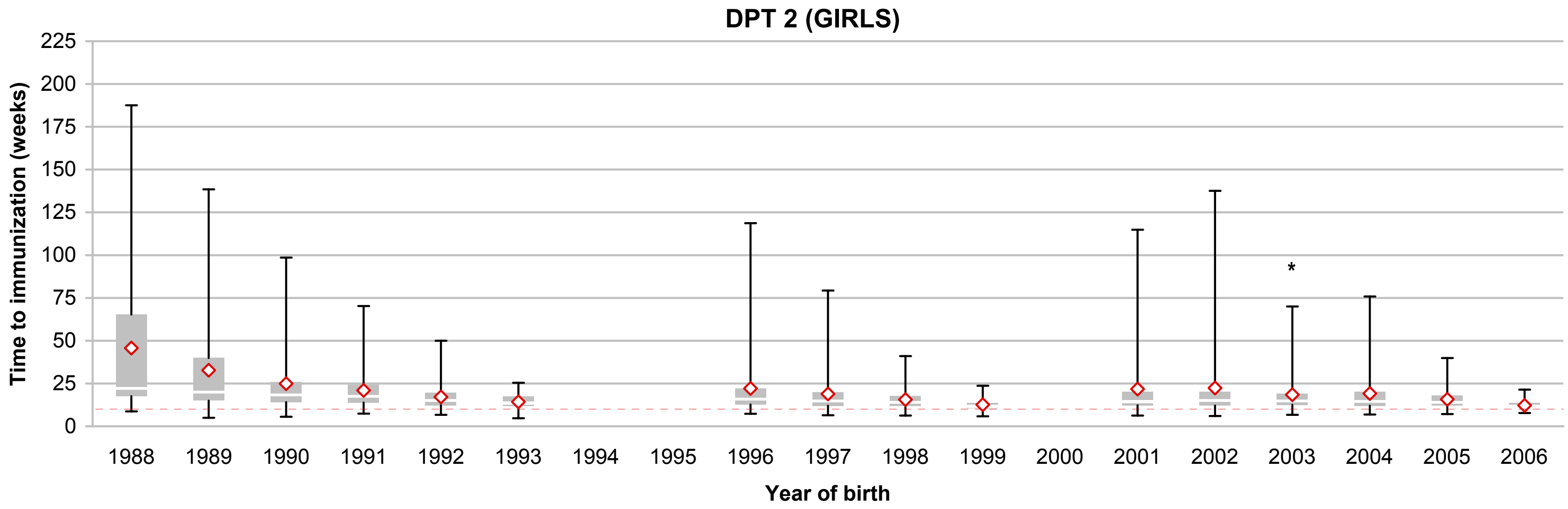

	1988	1989	1990	1991	1992	1993	1994	1995	1996	1997	1998	1999	2000	2001	2002	2003	2004	2005	2006
1st	8.71	5.00	5.57	7.43	6.71	4.71			7.29	6.43	6.29	5.86		6.29	6.00	6.71	6.86	7.14	7.71
25th	17.57	15.00	14.14	13.86	12.29	12.00			12.71	12.00	11.71	10.57		12.29	12.14	12.57	12.00	12.00	11.00
50th	22.14	19.86	18.29	17.57	15.14	13.57			16.00	14.86	14.00	11.71		14.43	15.00	14.86	14.57	13.86	11.43
75th	65.29	39.86	26.00	24.29	19.57	17.29			22.14	20.00	17.71	13.71		20.14	20.29	19.29	20.29	17.86	13.14
99th	187.57	138.43	98.57	70.29	50.00	25.43			118.71	79.29	41.00	23.71		114.86	137.57	70.00	75.86	39.86	21.43
Mean	45.69	32.77	24.94	20.95	17.19	14.31			22.11	18.89	15.63	12.65		21.75	22.39	18.46	19.12	15.75	12.20
SD	48.61	30.21	19.07	11.95	7.71	4.03			18.61	12.81	6.34	5.47		22.50	22.16	12.41	13.13	6.15	2.64
Minimum	8.71	3.71	3.00	2.86	4.14	4.71			4.00	3.14	0.00	5.00		5.14	2.43	1.29	4.14	3.86	6.43
Maximum	187.57	175.71	138.29	99.43	61.71	25.43			134.86	96.71	79.43	69.14		191.00	173.43	152.14	104.43	60.14	24.86
N	49	481	889	1334	1012	67			554	1110	1251	166		503	874	1033	1386	1608	156

* p <0.05 test for difference in mean time to vaccination between girls and boys

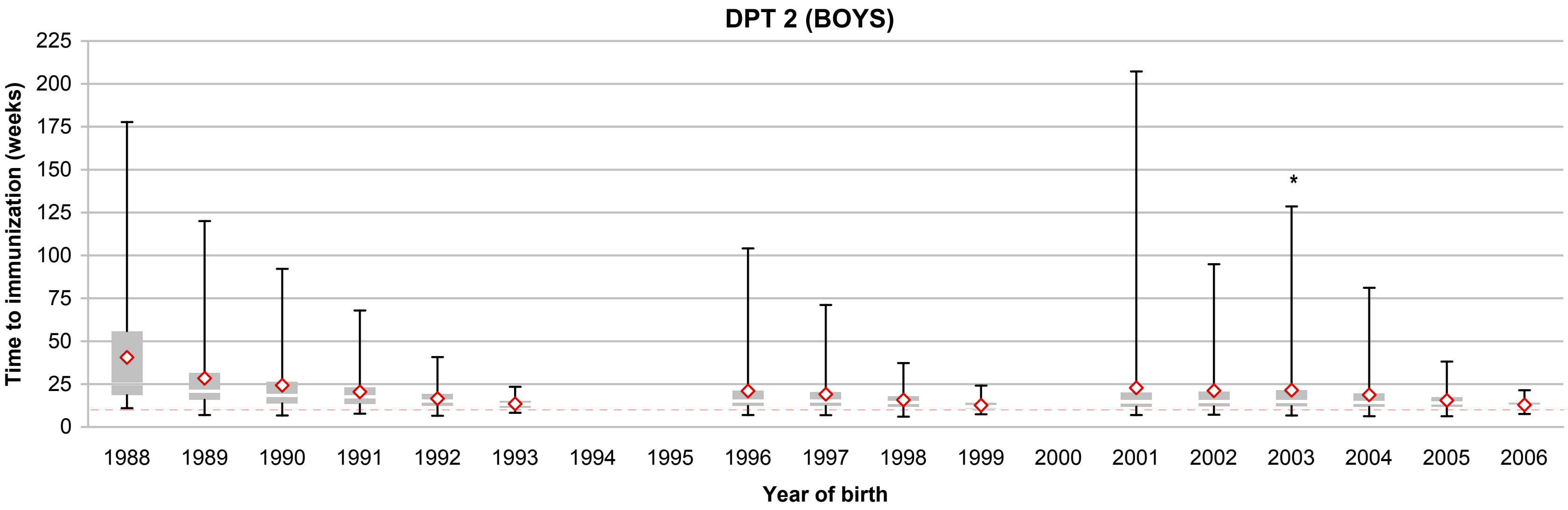

	1988	1989	1990	1991	1992	1993	1994	1995	1996	1997	1998	1999	2000	2001	2002	2003	2004	2005	2006
1st	11.00	7.00	6.71	7.71	6.57	8.29			7.00	6.86	6.00	7.43		7.00	7.14	6.71	6.29	6.29	7.57
25th	18.57	15.86	13.71	13.29	12.29	11.14			12.29	12.29	11.86	10.71		11.86	12.00	12.14	11.71	11.71	11.14
50th	25.00	20.71	18.29	17.43	15.00	13.14			15.14	15.14	14.29	11.86		14.71	14.71	14.86	14.29	13.86	12.14
75th	55.86	31.43	26.29	23.00	19.14	15.14			21.14	20.43	18.00	14.00		20.14	20.57	21.43	19.71	17.29	14.00
99th	177.71	120.00	92.14	67.86	40.71	23.43			104.14	71.14	37.29	24.14		207.29	94.86	128.57	81.14	38.14	21.43
Mean	40.54	28.34	24.30	20.45	16.55	13.51			20.94	19.08	15.77	12.66		22.83	21.11	21.38	18.69	15.52	12.89
SD	35.92	21.98	17.87	11.39	6.63	3.37			17.82	12.31	6.19	3.98		29.65	18.46	20.09	13.10	6.11	2.81
Minimum	11.00	3.43	2.14	1.00	3.71	6.71			4.71	3.29	1.14	6.43		6.29	0.71	1.14	4.43	1.71	5.57
Maximum	177.71	193.57	134.71	109.00	67.00	25.86			146.14	96.29	66.71	48.00		238.29	217.71	158.43	95.14	56.14	26.57
N	62	561	993	1503	1146	80			682	1299	1490	164		524	922	1278	1586	1852	173

* $p < 0.05$ test for difference in mean time to vaccination between girls and boys

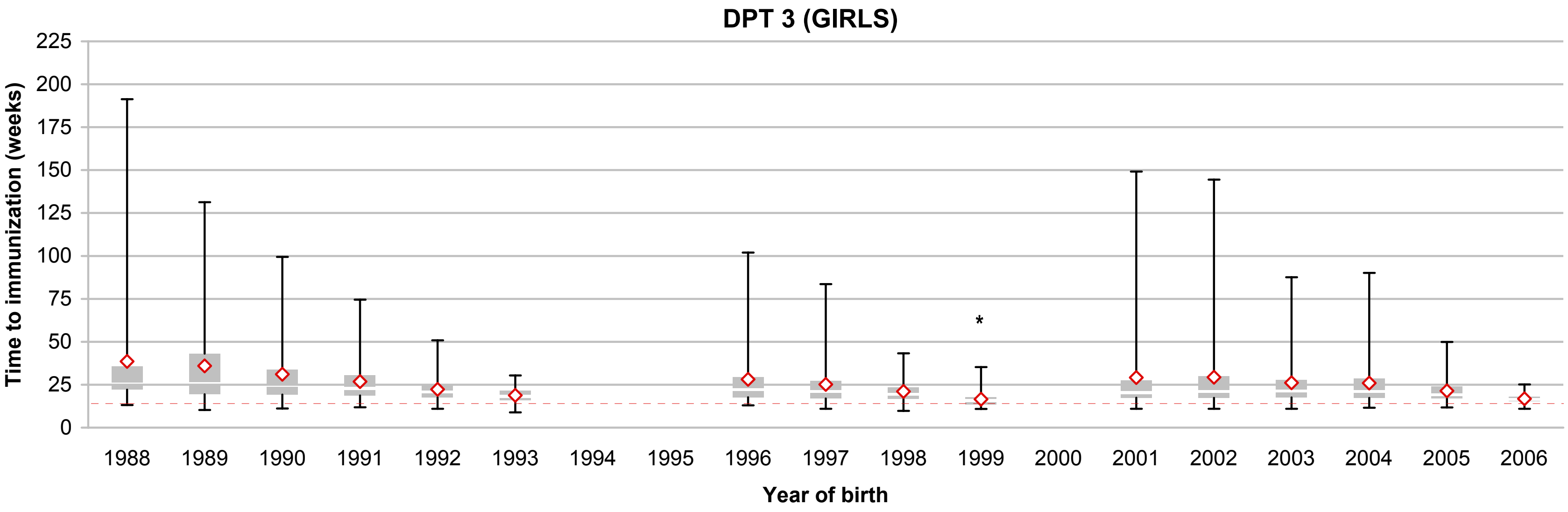

	1988	1989	1990	1991	1992	1993	1994	1995	1996	1997	1998	1999	2000	2001	2002	2003	2004	2005	2006
1st	13.14	10.29	11.14	11.86	11.00	8.86			13.00	11.00	9.71	10.86		11.00	11.00	11.00	11.57	11.71	11.00
25th	22.14	19.43	19.29	18.71	17.57	16.14			17.71	17.00	16.71	13.71		17.43	17.29	17.57	17.29	16.86	15.43
50th	25.43	25.86	24.57	22.86	20.86	18.14			22.29	21.00	19.43	15.71		20.43	21.00	21.29	21.00	19.14	16.43
75th	35.71	43.14	33.86	30.43	24.57	21.43			29.43	27.14	23.57	17.71		27.43	29.86	27.71	28.57	24.00	18.00
99th	191.29	131.29	99.43	74.57	50.86	30.43			102.00	83.57	43.29	35.29		149.14	144.43	87.57	90.14	49.86	25.14
Mean	38.60	35.99	31.12	26.78	22.27	18.79			28.08	25.19	21.08	16.52		29.18	29.36	26.09	25.93	21.42	16.85
SD	32.62	26.32	19.50	13.13	7.35	4.19			17.60	13.66	6.75	6.06		24.83	23.84	14.69	14.67	7.06	2.46
Minimum	13.14	9.43	8.86	6.29	8.29	8.86			9.57	8.43	5.00	8.71		7.14	5.00	6.29	5.57	7.86	10.86
Maximum	191.29	184.57	117.43	109.86	60.57	30.43			127.86	125.86	84.43	73.57		178.00	178.71	142.29	108.14	62.43	25.14
N	41	449	821	1215	768	28			519	1041	1019	80		494	841	984	1330	1340	77

* p <0.05 test for difference in mean time to vaccination between girls and boys

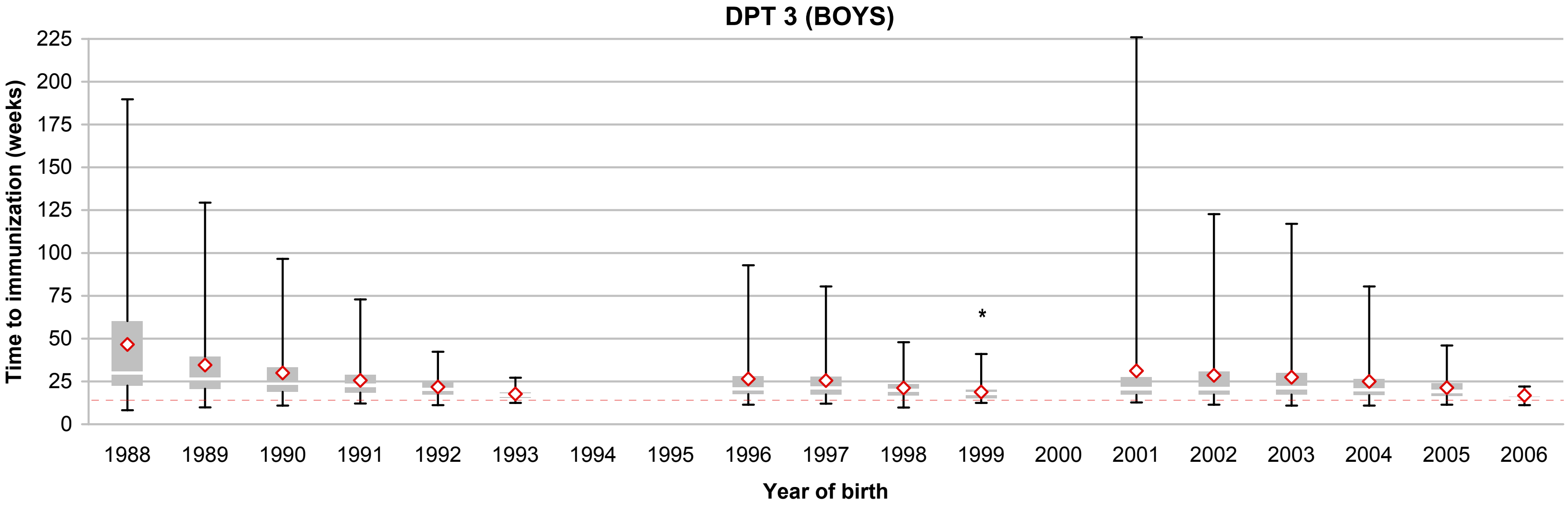

	1988	1989	1990	1991	1992	1993	1994	1995	1996	1997	1998	1999	2000	2001	2002	2003	2004	2005	2006
1st	8.14	9.86	10.86	12.14	11.14	12.43			11.43	12.00	9.71	12.43		12.71	11.43	10.86	10.86	11.43	11.14
25th	22.57	20.71	18.86	18.43	17.43	15.43			17.57	17.43	16.71	15.14		17.14	17.29	17.29	17.00	16.57	15.86
50th	30.00	26.29	24.00	22.71	20.43	17.00			20.71	21.29	19.71	17.86		20.71	20.86	21.29	20.14	19.14	17.00
75th	60.29	39.43	33.29	28.71	24.57	18.43			28.00	27.86	23.43	20.14		27.43	30.71	29.86	26.57	23.86	17.71
99th	189.71	129.43	96.57	72.86	42.29	27.14			92.86	80.43	47.86	41.00		226.00	122.71	117.00	80.43	46.00	22.00
Mean	46.60	34.56	29.99	25.61	21.76	17.66			26.44	25.50	21.16	18.77		31.19	28.58	27.41	24.97	21.30	16.85
SD	38.59	23.17	17.98	11.36	6.51	3.40			15.87	13.52	6.99	6.48		33.41	20.74	18.28	13.84	6.98	1.87
Minimum	8.14	4.29	7.14	0.29	0.71	8.00			10.29	9.29	5.86	7.00		1.29	5.57	1.71	6.86	6.71	9.71
Maximum	189.71	172.71	139.71	101.57	55.29	27.14			114.57	103.00	79.43	67.71		235.14	222.00	162.29	101.14	57.14	25.86
N	61	537	932	1372	881	43			638	1225	1226	86		507	893	1214	1497	1566	80

* p <0.05 test for difference in mean time to vaccination between girls and boys

MEASLES (GIRLS)

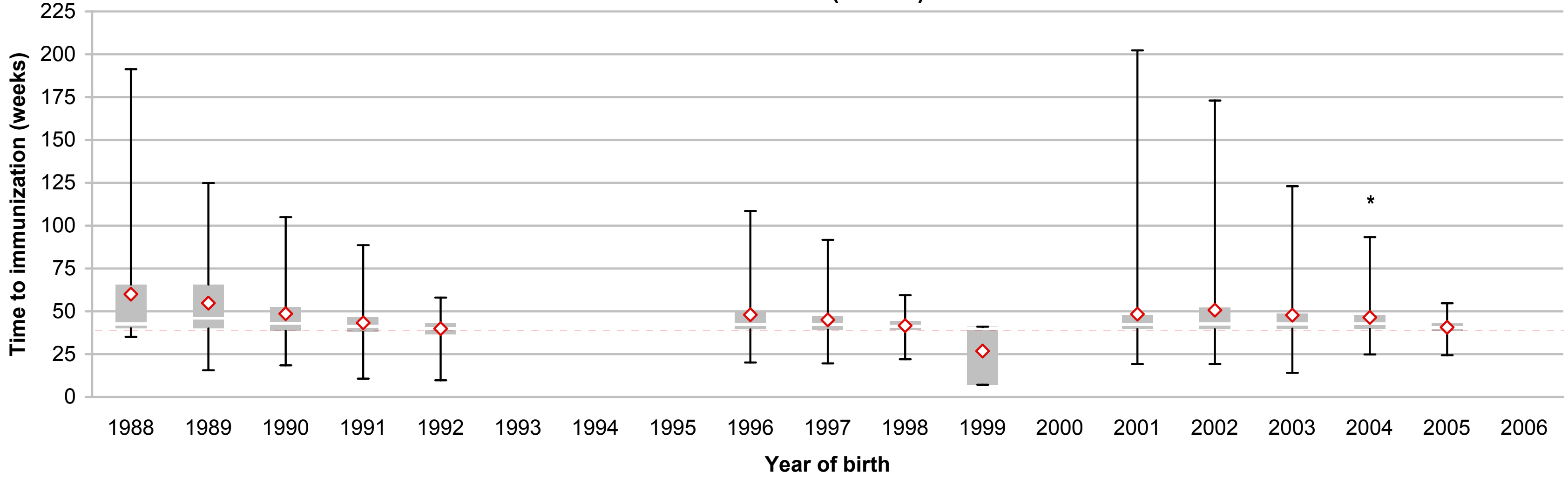

	1988	1989	1990	1991	1992	1993	1994	1995	1996	1997	1998	1999	2000	2001	2002	2003	2004	2005	2006
1st	35.14	15.57	18.43	10.71	9.71				20.14	19.57	22.00	7.14		19.29	19.29	14.14	24.86	24.43	
25th	40.14	40.00	38.71	38.00	36.57				39.43	39.29	39.14	7.14		39.71	39.57	39.86	39.71	38.57	
50th	42.71	46.00	43.00	41.43	39.86				42.29	42.43	41.14	39.86		42.29	42.71	42.57	42.71	40.57	
75th	65.57	65.57	52.43	46.86	43.29				49.57	47.29	44.29	41.00		47.86	52.14	48.57	47.86	43.00	
99th	191.29	124.86	105.00	88.57	58.00				108.57	91.71	59.43	41.00		202.29	173.00	123.00	93.29	54.71	
Mean	60.00	54.80	48.61	43.28	39.92				47.97	44.99	41.67	26.80		48.36	50.74	47.76	46.47	40.70	
SD	34.21	24.59	17.59	11.50	7.70				16.93	11.72	6.44	20.09		25.35	23.90	17.71	12.54	5.09	
Minimum	34.00	0.00	1.57	1.29	2.00				9.00	0.86	1.29	7.14		8.86	11.57	2.71	9.29	10.00	
Maximum	191.29	173.86	167.86	96.86	67.86				129.43	101.00	87.57	41.00		250.71	197.00	153.71	112.43	65.29	
N	34.00	370.00	658.00	873.00	277.00				444.00	893.00	441.00	3.00		459.00	789.00	913.00	1225.00	638.00	

* $p < 0.05$ test for difference in mean time to vaccination between girls and boys

MEASLES (BOYS)

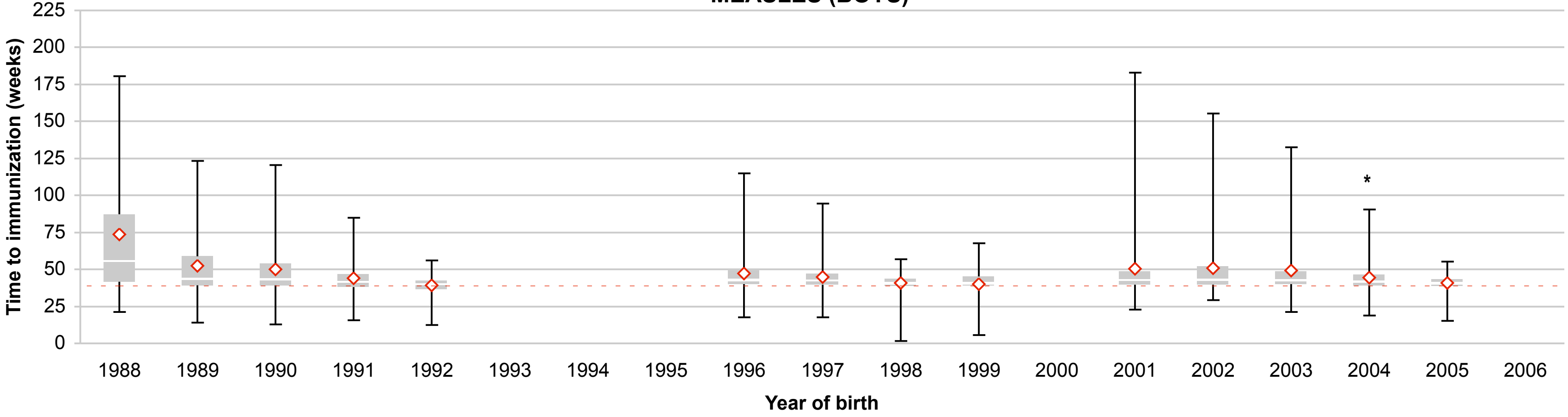

	1988	1989	1990	1991	1992	1993	1994	1995	1996	1997	1998	1999	2000	2001	2002	2003	2004	2005	2006
1st	21.14	13.86	12.71	15.43	12.29				17.43	17.57	1.57	5.57		22.86	29.14	21.14	18.71	15.29	
25th	41.57	39.14	39.14	38.00	36.43				39.86	39.57	38.86	39.43		39.57	39.57	39.86	39.29	39.00	
50th	55.71	43.71	43.29	41.57	40.00				42.71	42.43	40.71	40.57		42.86	42.86	42.71	41.71	40.71	
75th	87.29	59.00	54.00	47.00	42.57				49.14	47.43	43.71	45.29		48.86	51.86	48.86	46.57	43.14	
99th	180.71	123.43	120.43	84.86	56.00				114.86	94.29	56.71	67.71		183.00	155.14	132.43	90.43	55.14	
Mean	73.80	52.58	50.09	44.08	39.16				47.39	44.84	40.79	39.92		50.45	50.88	49.15	44.43	40.67	
SD	43.25	23.91	19.65	11.56	7.73				16.54	11.51	7.69	17.03		26.95	22.15	20.80	11.50	6.03	
Minimum	21.14	3.43	0.00	1.71	3.86				10.71	0.14	0.00	5.57		5.14	5.57	3.29	2.43	4.57	
Maximum	180.71	193.57	144.71	117.14	63.14				137.71	106.57	113.29	67.71		234.57	220.43	163.71	99.57	65.29	
N	42.00	441.00	759.00	1023.00	317.00				572.00	1027.00	533.00	9.00		465.00	821.00	1129.00	1396.00	763.00	

* p <0.05 test for difference in mean time to vaccination between girls and boys
